# Efficient pulmonary lymphatic drainage is necessary for inflammation resolution in ARDS

**DOI:** 10.1172/jci.insight.173440

**Published:** 2024-01-09

**Authors:** Pu-hong Zhang, Wen-wu Zhang, Shun-shun Wang, Cheng-hua Wu, Yang-dong Ding, Xin-yi Wu, Fang Gao Smith, Yu Hao, Sheng-wei Jin

**Affiliations:** 1 Department of Anaesthesia and Critical Care, the Second Affiliated Hospital and Yuying Children’s Hospital of Wenzhou Medical University, Zhejiang, China.; 2 Key Laboratory of Pediatric Anesthesiology, Ministry of Education, Wenzhou Medical University, Zhejiang, China.; 3 Key Laboratory of Anesthesiology of Zhejiang Province, the Second Affiliated Hospital and Yuying Children’s Hospital of Wenzhou Medical University, Zhejiang, China.; 4 Academic Department of Anesthesia, Critical Care, Resuscitation and Pain, Heart of England NHS Foundation Trust, Birmingham, United Kingdom.

**Keywords:** Pulmonology, Vascular Biology, Respiration

## Abstract

The lymphatic vasculature is the natural pathway for the resolution of inflammation, yet the role of pulmonary lymphatic drainage function in sepsis-induced acute respiratory distress syndrome (ARDS) remains poorly characterized. In this study, indocyanine green–near infrared lymphatic living imaging was performed to examine pulmonary lymphatic drainage function in septic mouse models. We found that the pulmonary lymphatic drainage was impaired owing to the damaged lymphatic structure in sepsis-induced ARDS. Moreover, prior lymphatic defects by blocking vascular endothelial growth factor receptor-3 (VEGFR-3) worsened sepsis-induced lymphatic dysfunction and inflammation. Posttreatment with vascular endothelial growth factor-C (Cys156Ser) (VEGF-C156S), a ligand of VEGFR-3, ameliorated lymphatic drainage by rejuvenating lymphatics to reduce the pulmonary edema and promote draining of pulmonary macrophages and neutrophils to pretracheal lymph nodes. Meanwhile, VEGF-C156S posttreatment reversed sepsis-inhibited CC chemokine ligand 21 (CCL21), which colocalizes with pulmonary lymphatic vessels. Furthermore, the advantages of VEGF-C156S on the drainage of inflammatory cells and edema fluid were abolished by blocking VEGFR-3 or CCL21. These results suggest that efficient pulmonary lymphatic drainage is necessary for inflammation resolution in ARDS. Our findings offer a therapeutic approach to sepsis-induced ARDS by promoting lymphatic drainage function.

## Abstract 2

The resolution of inflammation in sepsis-induced acute respiratory distress syndrome requires efficient lymphatic drainage in the lung.

## Graphical abstract

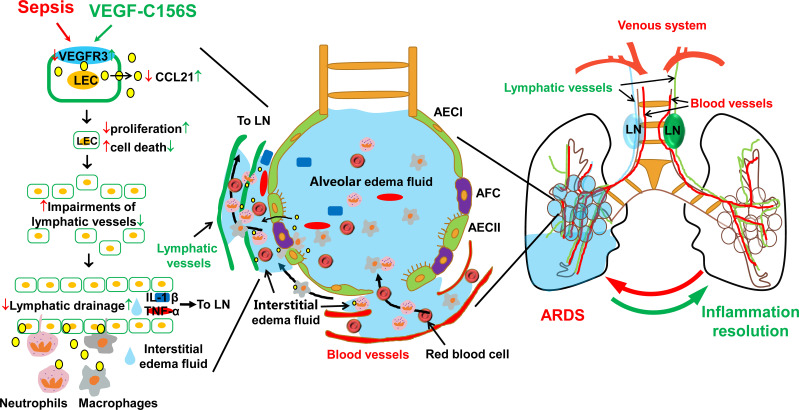

## Introduction

Sepsis is an infection-induced critical illness characterized by multiple organ dysfunctions with unacceptably high mortality and often accompanies the serious complication of acute respiratory distress syndrome (ARDS) (1). The main pathogenic factors of sepsis-induced ARDS are the accumulation of alveolus and interstitial inflammatory cells and edema fluid that lead to hypoxemia and hypercapnia (2). Unfortunately, antiinflammatory therapies from almost all clinical trials have not been shown to reduce mortality (2–4). Moderate inflammation is an inherently protective physiological response to the infection and intimately involved in the restoration of tissue homeostasis, while the resolution of inflammation is an active and orchestrated process (5–7). To remove excess inflammatory cells and edema fluid timely and effectively is essential in ARDS recovery (2, 8). Conversely, the delay of inflammation resolution causes and aggravates ARDS (5, 9, 10). So, how to promote the resolution of inflammation is being recognized as an important issue for recovery from sepsis-induced ARDS.

The lymphatic vasculature is the natural route for the resolution of inflammation (11, 12). The lymphatic vessels transport immune cells and protein-rich interstitial fluid to prevent tissue edema and facilitate immunomodulation and immunosurveillance (12–14). The lymphatic vessels are lined with the lymphatic endothelial cells (LECs), characterized by the distinct expression of lymphatic vessel endothelial hyaluronan receptor 1 (LYVE1), homeobox transcription factor Prospero-related homeobox-1 (Prox1), podoplanin, and vascular endothelial growth factor receptor-3 (VEGFR-3) (11, 12). Furthermore, VEGF-C is a ligand of VEGFR-3, and its functions in developmental, pathological, and therapeutic lymphangiogenesis are achieved through VEGFR-3–mediated signaling pathways (15–18). Recent studies of the lymphatic vasculature mainly highlight molecular and structural features that contribute to controlling disease severity in the derma (19, 20), heart (17, 21), aorta (12), meninges (13, 22, 23), mesentery (24), and liver (25, 26).

Pulmonary lymphatic vessels are abundantly distributed along with blood vessels and bronchial tubes in pulmonary interstitium and also are necessary for the development of neonatal lungs (27, 28). Lymphatic impairment leads to an inflammatory state characterized by the formation of tertiary lymphoid organs, which participate in regulating immune tolerance in lung allografts (27, 29). In addition, pulmonary fibrosis impairs lymphatic drainage function, while lymphatic proliferation ameliorates macrophage accumulation and fibrosis (30, 31). However, the role of pulmonary lymphatics in sepsis-induced ARDS is less well researched.

Here, we sought to determine the pulmonary lymphatic drainage function using living lymphatic imaging technology in real time to explore 1) how the pulmonary lymphatic function is impaired by sepsis and the specific mechanism therein and 2) whether improving pulmonary lymphatic function could result in the resolution of pulmonary inflammation in sepsis-induced ARDS.

## Results

### Sepsis causes the lymphatic drainage dysfunction.

Sepsis is a systemic disease that often accompanies progressive subcutaneous edema (32). In order to determine whether lymphatic drainage function was affected by sepsis, we observed the dermal lymphatic drainage function in different-dose LPS-induced sepsis mouse models. The lymphatic drainage function was detected by real-time indocyanine green–near infrared lymphatic (ICG-NIR) lymphatic living imaging using an in vivo imaging system (IVIS) (Supplemental Figure 2, A and B; supplemental material available online with this article; https://doi.org/10.1172/jci.insight.173440DS1). We detected that the fluorescence intensities of the footpad injection site were significantly higher in LPS-induced (10 mg/kg) sepsis mice at the 24th hour and 48th hour (Supplemental Figure 2, C and D). In addition, LPS-induced (10 mg/kg) sepsis mice had lower ICG clearance rate at the 24th hour and 48th hour, while no difference was observed in the low-dose LPS (1 mg/kg) group (Supplemental Figure 2, C–F). These data suggested that the high dose of LPS (10 mg/kg), rather than the low dose of LPS (1 mg/kg), could lead to impairments of the dermal lymphatic drainage function. Thus, the high-dose LPS-induced (10 mg/kg) sepsis model was used in the following experiments.

Next, at the 24th hour, the fluorescence intensities of ICG in the draining popliteal lymph nodes (dpLNs) were determined using IVIS and confocal microscopy at 30 minutes after the ICG injection. We observed a significant decrease of fluorescence intensities in dpLNs in sepsis mice (Supplemental Figure 2, G and H). Furthermore, similar results have also been shown in the ear skin (Supplemental Figure 2, I–N) in sepsis mice. We also detected significant lymphatic blockage in sepsis mice (Supplemental Videos 1 and 2). These data suggested that the sepsis produced a systemic lymphatic drainage dysfunction.

**Figure 1:**
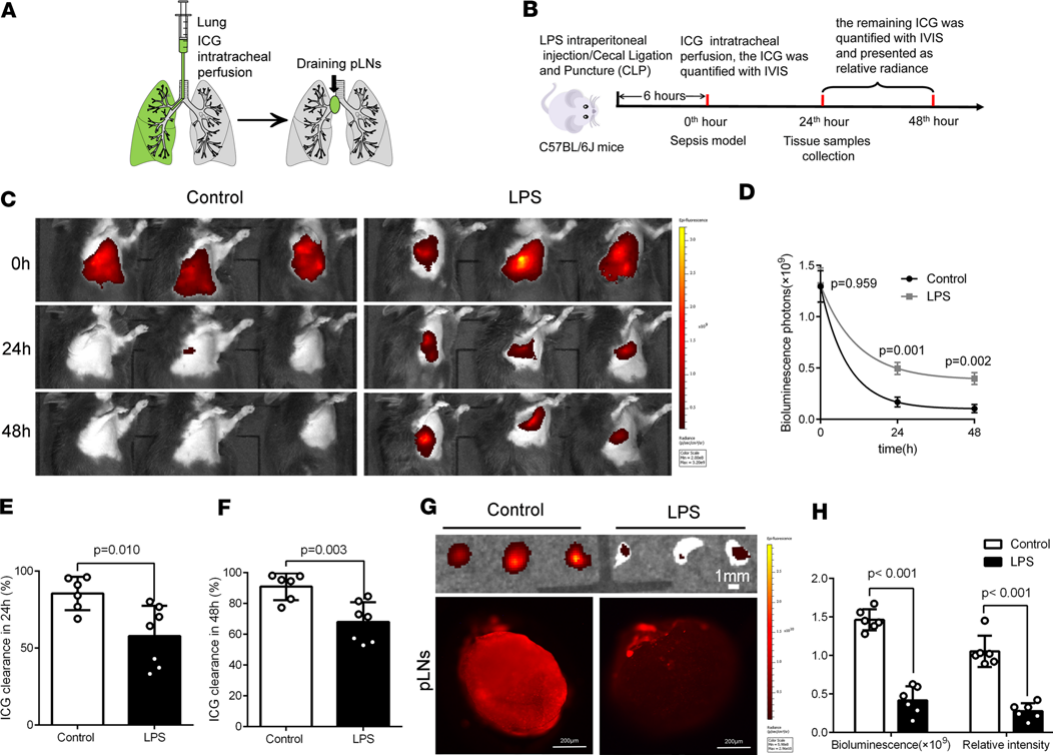
The pulmonary lymphatic drainage function is impaired in an LPS-induced sepsis model. (**A**) ICG was intratracheally poured into unilateral lung, and ICG drained from the pulmonary alveoli into the pretracheal lymph nodes (pLNs). (**B**) Procedure and timeline: At 6 hours after the intraperitoneal injection of LPS (10 mg/kg) or the CLP, the sepsis model was induced. ICG (1 mg/mL, 10 μL) was intratracheally poured into unilateral lung. Fluorescence intensities of ICG were determined at 0 hours, 24th hour, and 48th hour using an IVIS associated with the clearance rate to reflect the effect of tissue fluid clearance by lymphatic flow. The lung tissue samples were collected at the 24th hour. (**C** and **D**) ICG was intratracheally poured into unilateral lung and quantified and presented as relative radiance (photons/s per cm^2^ /steradian) at 0 hours, 24th hour, and 48th hour using IVIS in LPS-induced sepsis model. (**E** and **F**) The ICG clearance rate in 24th hour and 48th hour (control *n* = 6, LPS *n* = 7; representative data from 3 independent experiments). (**G** and **H**) Fluorescence intensities of ICG were determined in pLNs at 30 minutes after the pour of ICG by IVIS and confocal microscopy in LPS-induced sepsis model at the 24th hour (control *n* = 6, LPS *n* = 6; representative data from 3 independent experiments). All *n* values refer to the number of mice used, and the error bars depict mean ± SD. *P* values were calculated by 2-tailed paired or unpaired Student’s *t* test.

**Figure 2:**
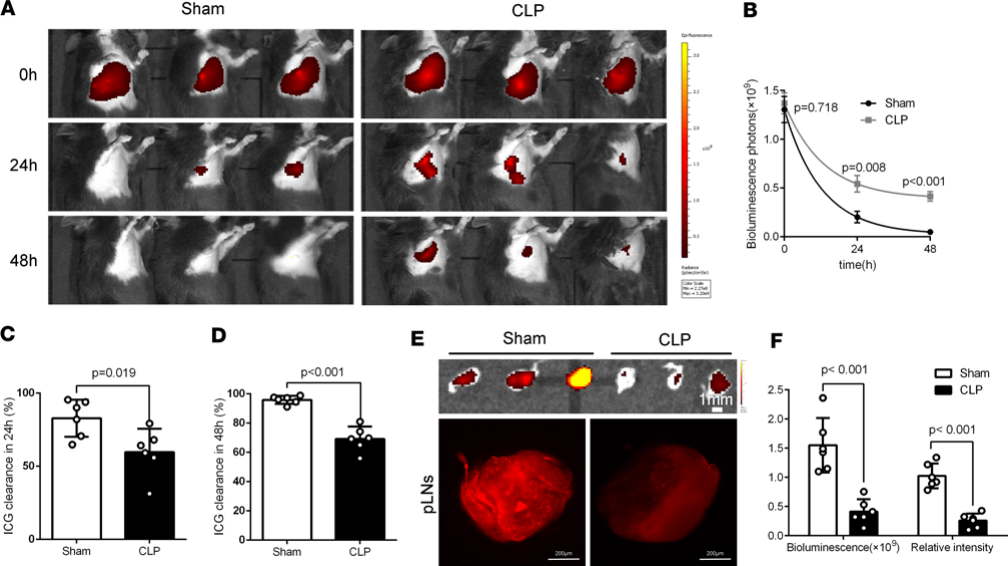
The pulmonary lymphatic drainage function is impaired in a CLP-induced sepsis model. (**A** and **B**) ICG was intratracheally poured into unilateral lung and quantified and presented at 0 hours, 24th hour, and 48th hour using IVIS in CLP-induced sepsis model. (**C** and **D**) The ICG clearance rate at 24th hour and 48th hour (sham *n* = 6, CLP *n* = 6; representative data from 3 independent experiments). (**E** and **F**) Fluorescence intensities of ICG were determined in pLNs at 30 minutes after the pour of ICG by IVIS and confocal microscopy in CLP-induced sepsis model at 24th hour (sham *n* = 6, CLP *n* = 6; representative data from 3 independent experiments). All *n* values refer to the number of mice used, and the error bars depict mean ± SD. *P* values were calculated by 2-tailed paired or unpaired Student’s *t* test.

Sepsis causes the pulmonary edema that leads to the serious complication of ARDS (1). To further investigate the role of pulmonary lymphatic drainage during sepsis-induced ARDS, we examined the residual ICG in lung and the draining ICG in pretracheal lymph nodes (pLNs) in LPS/cecal ligation and puncture–induced (CLP-induced) sepsis mice (Figure 1A). Sepsis mice had markedly higher fluorescence intensities of residual ICG associated with lower ICG clearance rate at the 24th hour and 48th hour in lungs (Figure 1, C–F, and Figure 2, A–D), as well as lower fluorescence intensities of draining ICG in pLNs (Figure 1, G and H, and Figure 2, E and F). We measured lung lymphatic drainage by another lymphatic tracer, FITC-labeled dextran, and detected similar results (Supplemental Figure 18). These findings further verified that sepsis also caused the impairments of pulmonary lymphatic drainage function.

### Sepsis induces structural damage of lymphatic vessels.

**Figure 3:**
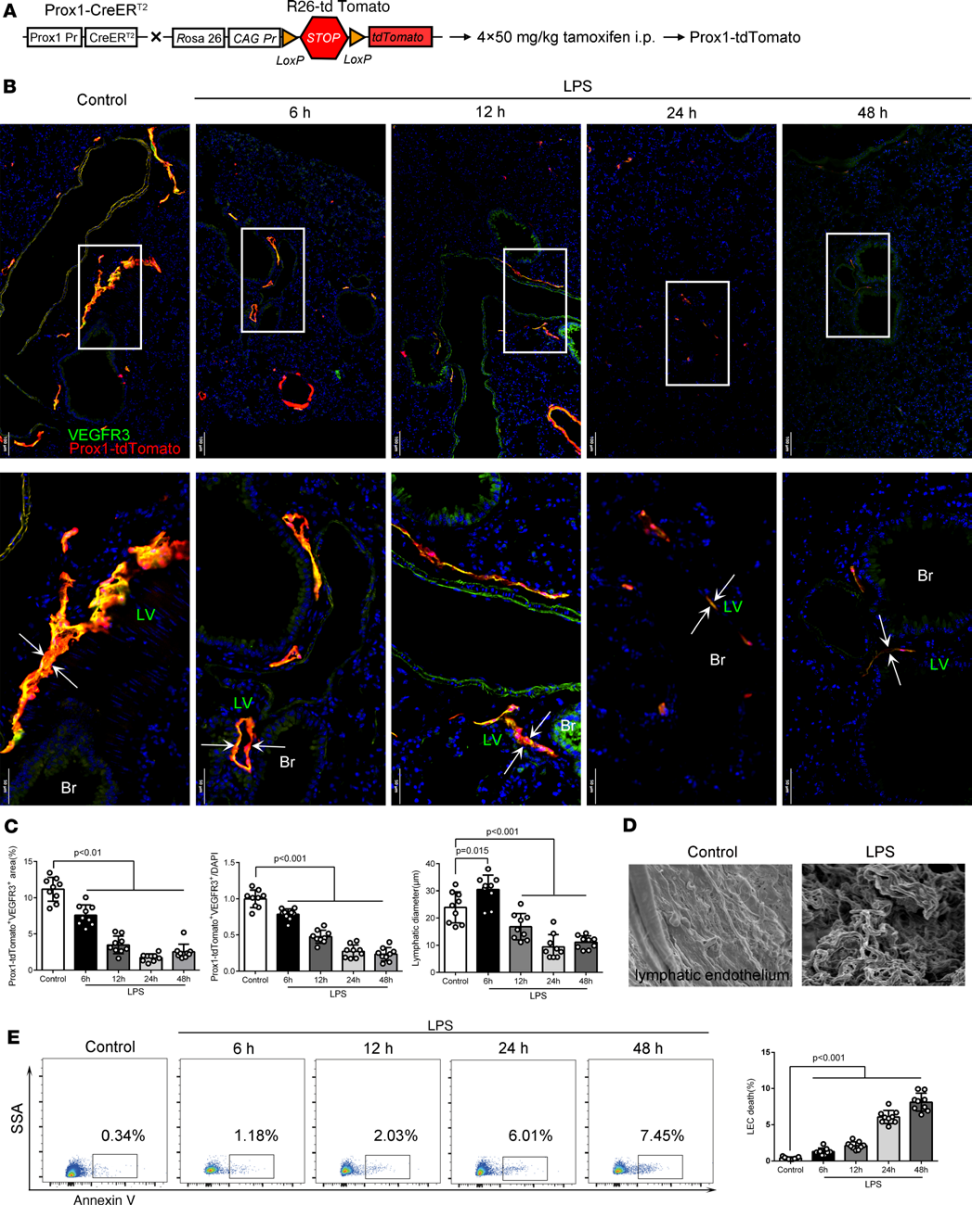
The pulmonary lymphatic vessels are damaged in an LPS-induced sepsis model. (**A**) Schematic representation of a conditional, lymph-specific fluorescence mouse model (*Prox1-CreER^T2+^ Rosa26-tdTomato^+^*). *Prox1-CreER^T2^* mice were crossed with *Rosa26-tdTomato* mice and treated with tamoxifen for 4 consecutive days before initiation of sepsis studies. (**B**) The pulmonary lymphatic vessels were labeled with an immunofluorescence stain of VEGFR-3 (green) and Prox1-tdTomato (red) signals from *Prox1-CreER^T2+^ R26-tdTomato^+^* mice after formation of sepsis. Scale bars, 100 μm (top), 50 μm (bottom). (**C**) Quantification of the percentage area coverage, the relative fluorescence intensity, and the diameter of lymphatic vessels (*n* = 9 per group; representative data from 3 independent experiments). (**D**) Scanning electron microscopy of the pulmonary lymphatic endothelium. Scale bars, 1 μm. (**E**) Representative flow cytometry images and the quantification of dead lymphatic endothelial cells (LECs) from lung suspension. The frame showed the percentage of annexin V^+^ LECs. (*n* = 9 per group; representative data from 3 independent experiments.) All *n* values refer to the number of mice used, and the error bars depict mean ± SD. *P* values were calculated by a 1-way ANOVA with Tukey’s multiple-comparison test.

To observe the pathological changes of pulmonary lymphatic vessels during sepsis, we used inducible Prox1^+^ LEC-specific lineage-tracing *Prox1-CreER^T2+^* *Rosa26-tdTomato^+^* mice (23, 33) (Figure 3A). We examined pulmonary lymphatic vessels marked with Prox1-tdTomato (red) and VEGFR-3 immunofluorescence (green) at 6 hours, 12 hours, 24 hours, and 48 hours in LPS-induced sepsis mice and found that the diameter of pulmonary lymphatic vessels was increased at 6 hours and decreased subsequently (Figure 3, B and C). Additionally, the percentage area coverage and the relative intensity of pulmonary lymphatic vessels (Prox1-tdTomato^+^VEGFR-3^+^/Prox1-tdTomato^+^CD31^+^/Prox1^+^) were overall decreased in LPS/CLP-induced sepsis mice (Figure 3, B and C; Supplemental Figure 3, B and D; Supplemental Figure 4, A and B; and Supplemental Figure 15), especially at 24 hours and 48 hours (Figure 3, B and C). Ultrastructurally, sepsis mice showed significant breakage of the integrity of pulmonary lymphatic endothelium (Figure 3D, Supplemental Figure 4C, and Supplemental Figure 19). Furthermore, LPS-induced sepsis mice had fewer dermal lymphatic vessels (Supplemental Figure 6, A–C) and the disruption of the lymphatic endothelial barrier (Supplemental Figure 6D). Furthermore, we also detected a significant reduction of pulmonary blood vessels (CD31^+^Prox1-tdTomato^–^) (Supplemental Figure 3, B and C) and the disruption of vascular endothelial barrier (Supplemental Figure 3A) in LPS-induced sepsis mice.

To investigate the mechanism of lymphatic injury during sepsis, we assessed the LECs’ death and proliferation. Notably, sepsis mice showed increased apoptosis (Supplemental Figure 5A, Supplemental Figure 20, and Figure 3E) and decreased proliferation of pulmonary LECs in vivo (Supplemental Figure 5B). These data indicated that increased death and inhibited proliferation of LECs may be associated with lymphatic injury during sepsis.

Besides that, sepsis also led to severe tissue edema; increases in accumulation of inflammatory cells, mainly macrophages and neutrophils, and of inflammatory factors; as well as severe tissue damage simultaneously in the lung (Supplemental Figure 4, D–I, and Supplemental Figure 7) and the skin (Supplemental Figure 6E). Overall, these results indicated that sepsis induced serious structural damage of lymphatic vessels and severe inflammatory response.

### Posttreatment with VEGF-C156S improves lymphatic drainage by rejuvenating lymphatics in sepsis.

**Figure 4:**
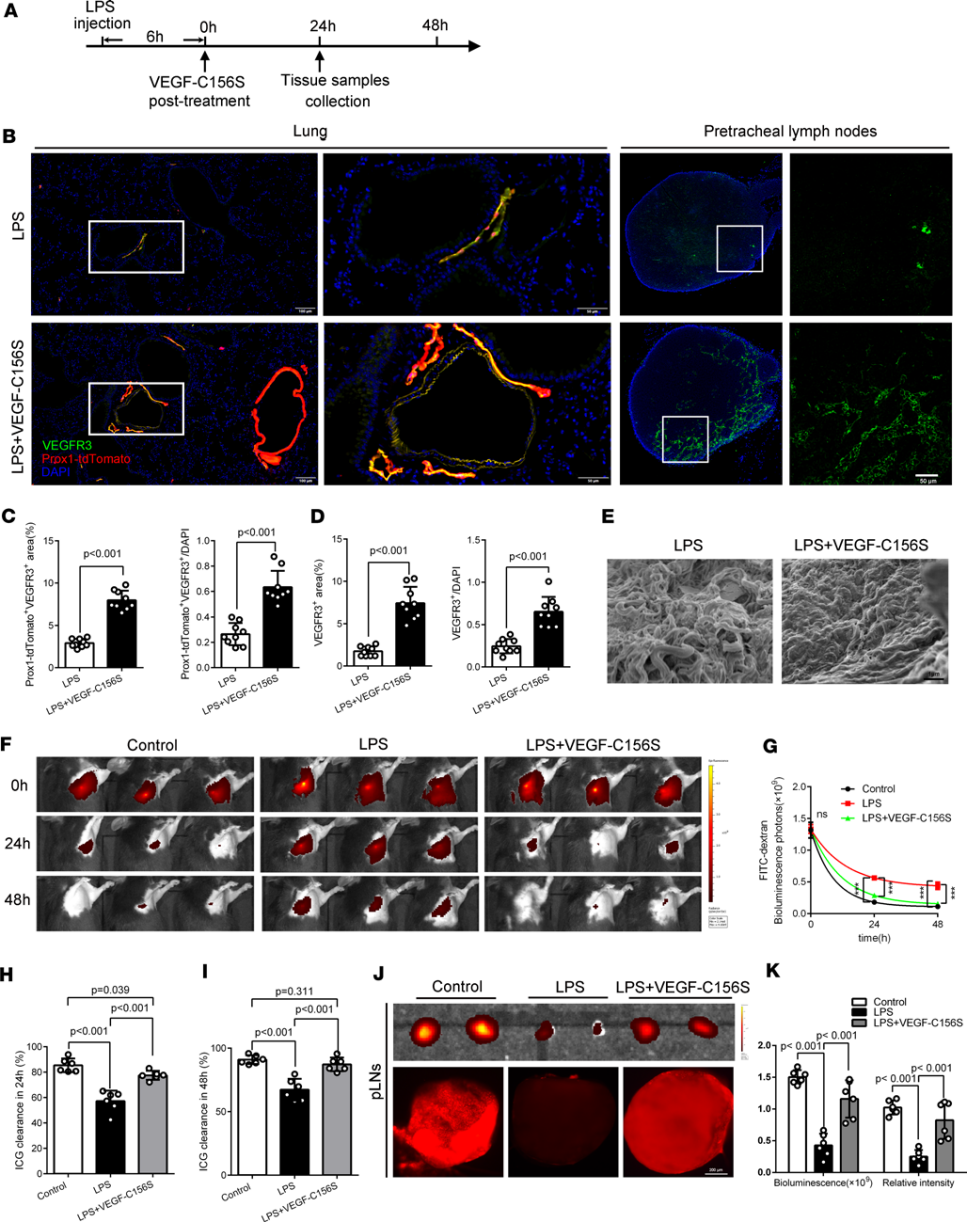
Posttreatment with VEGF-C156S ameliorated pulmonary lymphatic drainage function by rejuvenating lymphatics in LPS-induced sepsis. (**A**) Procedure and timeline: Recombinant VEGF-C156S protein was administrated to an LPS-induced sepsis model 6 hours afterward. Then, the lung tissue and the pLNs were obtained at the 24th hour. (**B**) Lymphatic vessels were labeled with an immunofluorescence stain of VEGFR-3 (green) and Prox1-tdTomato signals (red). Scale bars for lung, 100 μm (left), 50 μm (right). Scale bar for pLNs, 50 μm. (**C**) Quantification of the percentage area coverage and the relative fluorescence intensity of lymphatic vessels in lungs (LPS = 9, LPS + VEGF-C156S = 9; representative data from 3 independent experiments). (**D**) Quantification of the percentage area coverage and the relative fluorescence intensity of lymphatic vessels in pLNs (LPS = 9, LPS + VEGF-C156S = 9; representative data from 3 independent experiments). (**E**) Scanning electron microscopy of the pulmonary lymphatic endothelium. Scale bar, 1 μm. (**F** and **G**) ICG was intratracheally poured into unilateral lung and quantified and presented as relative radiance at 0 hours, 24th hour, and 48th hour using IVIS. (**H** and **I**) The ICG clearance rate at the 24th hour and 48th hour (control *n* = 6, LPS *n* = 6, LPS + VEGF-C156S *n* = 6; representative data from 3 independent experiments). (**J** and **K**) At the 24th hour, ICG was intratracheally poured into unilateral lung. Fluorescence intensities of ICG were determined in pLNs at 30 minutes after the pour by IVIS and confocal microscopy (control *n* = 6, LPS *n* = 6, LPS + VEGF-C156S *n* = 6; representative data from 3 independent experiments). Scale bar, 200 μm. All *n* values refer to the number of mice used, and the error bars depict mean ± SD. *P* values were calculated by a 1-way ANOVA with Tukey’s multiple-comparison test.

Recent research showed that vascular endothelial growth factor-C (Cys156Ser) (VEGF-C156S) could induce VEGFR-3–mediated lymphangiogenesis in pathological conditions (17, 18). Our data showed that the area of lymphatic vessels was significantly reduced by sepsis, so we wondered whether VEGF-C156S could promote lymphangiogenesis in sepsis. Indeed, VEGF-C156S posttreatment reduced LECs’ apoptosis and increased their proliferation in LPS-induced sepsis mice (Supplemental Figure 5, A and B). Moreover, VEGF-C156S posttreatment increased the percentage area and the relative fluorescence intensities of pulmonary/LN lymphatic vessels (Prox1-tdTomato^+^VEGFR-3^+^/Prox1-tdTomato^+^CD31^+^) (Figure 4, B–D, and Supplemental Figure 8, B and C) and endothelial integrity (Supplemental Figure 19) in LPS/CLP-induced sepsis mice. In addition, dermal lymphatic vessels also showed similar responses by posttreatment of VEGF-C156S (Supplemental Figure 9, A–C). Next, we found that the falloff and disruption of lymphatic endothelium were obviously ameliorated by posttreatment with VEGF-C156S in sepsis (Figure 4E and Supplemental Figure 4E). In addition, VEGF-C156S posttreatment reduced the lymphangial exudation of Evans blue in LPS-induced sepsis mice (Supplemental Figure 9D), suggesting that VEGF-C156S could repair the damaged lymphatic vessels and rescue barrier function. Overall, these data indicated that VEGF-C156S posttreatment could rejuvenate and restore pulmonary lymphatics in sepsis.

Then, we found that VEGF-C156S posttreatment accelerated the drainage of ICG/FITC-dextran from the lung into the pLNs in LPS/CLP-induced sepsis mice (Figure 4, F–K; Supplemental Figure 8, F–K; and Supplemental Figure 18), indicating that VEGF-C156S–induced lymphatics rejuvenation could ameliorate the pulmonary lymphatic drainage during sepsis. Likewise, similar effects existed in the ear and footpad of LPS-induced sepsis mice (Supplemental Figure 10, A–J). Moreover, we detected that VEGF-C156S posttreatment could significantly increase the speed of lymphatic flow (Supplemental Figure 10, K–M). Taken together, these results indicated that the lymphatic rejuvenation generated by VEGF-C156S posttreatment could improve pulmonary lymphatic drainage.

### Improvement of lymphatic drainage promotes the resolution of inflammation by accelerating the inflammatory cells draining to pLNs.

**Figure 5:**
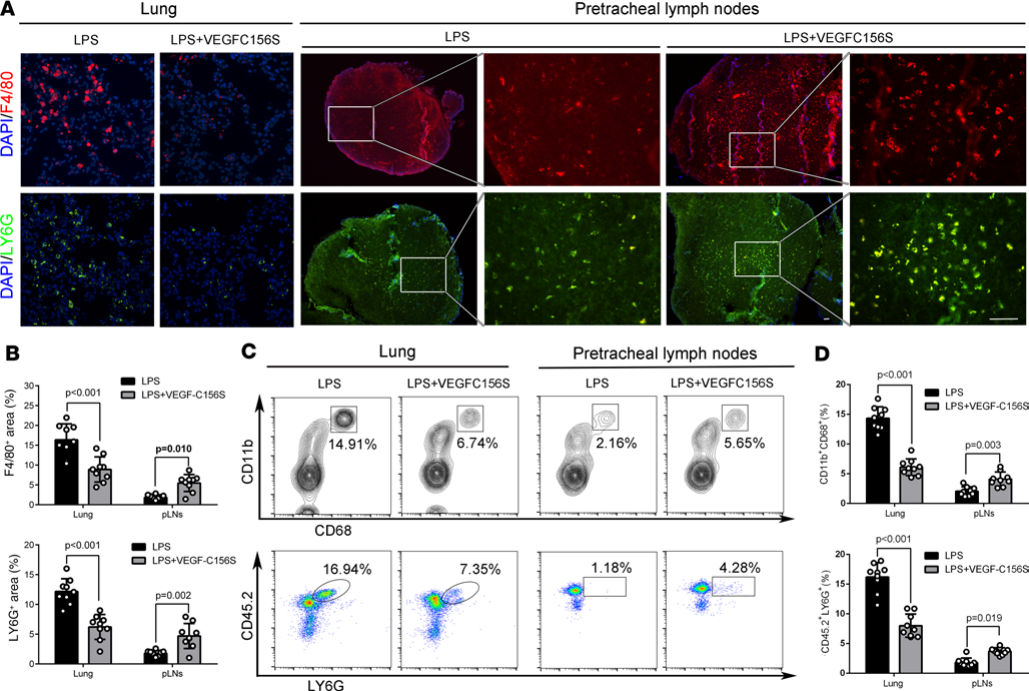
VEGF-C156S posttreatment promoted pulmonary inflammatory cells draining to pLNs in LPS-induced sepsis. Recombinant VEGF-C156S protein was administrated to LPS-induced sepsis model. Then, the lung tissue and the pLNs were obtained at the 24th hour. (**A** and **B**) Representative immunofluorescence images and quantification of F4/80^+^ cells (red, macrophages) and LY6G^+^ cells (green, neutrophil) in lung sections and pLN sections (LPS = 9, LPS + VEGF-C156S = 9; representative data from 3 independent experiments). Scale bars, 50 µm. (**C** and **D**) Representative flow cytometry images and quantification of F4/80^+^/CD45.2^+^ cells (macrophages) and LY6G^+^ cells (neutrophils) in lung tissue and pLNs. The frame showed the percentage of F4/80^+^/CD45.2^+^ cells or LY6G^+^ cells. (LPS = 9, LPS + VEGF-C156S = 9; representative data from 3 independent experiments). All *n* values refer to the number of mice used, and the error bars depict mean ± SD. *P* values were calculated by 2-tailed unpaired Student’s *t* test.

**Figure 6:**
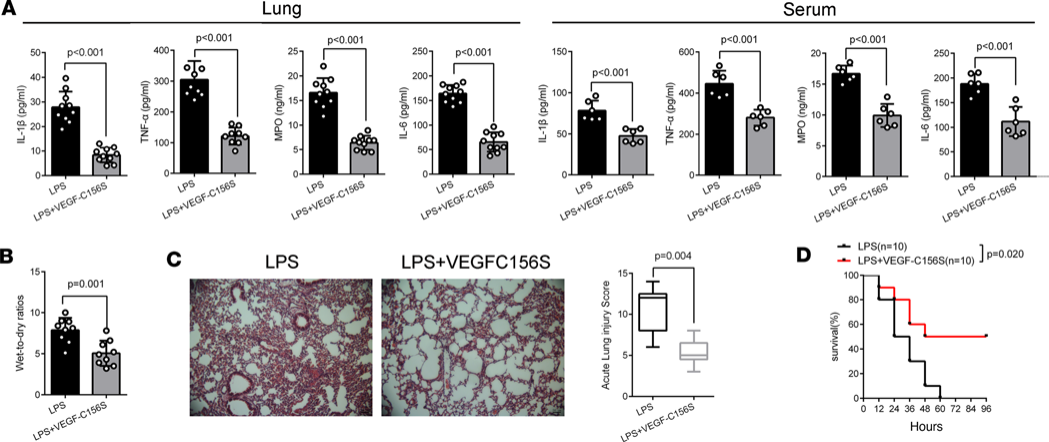
VEGF-C156S posttreatment promotes pulmonary inflammation resolution in LPS-induced sepsis. (**A**) The concentrations of inflammatory factors in lung tissue homogenate or the serum, such as IL-1β, TNF-α, MPO, and IL-6, were measured by ELISA (*n* = 9~10; representative data from 3 independent experiments). (**B**) Wet-to-dry ratios for lungs (LPS = 9, LPS + VEGF-C156S = 9; representative data from 3 independent experiments). (**C**) Representative images of lung H&E-stained sections and the acute lung injury scores (LPS = 9, LPS + VEGF-C156S = 9; representative data from 3 independent experiments). Box plots show the interquartile range (box), median (line), and minimum and maximum (whiskers). (**D**) The survival curve. Mice received a single intraperitoneal injection with a lethal dose of LPS (40 mg/kg of body weight), followed by a single tail vein injection of VEGF-C156S (0.1 μg/g of body weight, 6 hours apart; LPS *n* = 10, LPS + VEGF-C156S *n* = 10). All *n* values refer to the number of mice used, and the error bars depict mean ± SD. *P* values were calculated by 2-tailed unpaired Student’s *t* test. The lung injury scores were shown as median (quartile) [M (P25, P75)] and analyzed by using the Kruskal-Wallis test. The survival rate in each subgroup was estimated by Kaplan-Meier survival curves and compared by the pairwise log-rank test.

VEGF-C156S posttreatment also increased the percentage area and the relative fluorescence intensities of lymphatics in the pLNs in LPS/CLP-induced sepsis (Figure 4, B and D, and Supplemental Figure 8, B and D) and reduced the accumulation of macrophages (F4/80^+^CD45.2^+^ cells or CD68^+^ cells) and neutrophils (LY6G^+^ cells) in the lung, while increasing these cells in pLNs (Figure 5, Supplemental Figure 11, and Supplemental Figure 12, A–C). In addition, VEGF-C156S posttreatment decreased inflammatory factor levels (Figure 6A and Supplemental Figure 12D), lung wet-to-dry ratios (Figure 6B and Supplemental Figure 12E), lung injury scores (Figure 6C and Supplemental Figure 12F), and the swelling of skin (Supplemental Figure 9E), demonstrating that VEGF-C156S–mediated ameliorative lymphatic drainage could attenuate tissue edema and damage. Moreover, VEGF-C156S posttreatment significantly increased the survival rates of LPS/CLP-induced sepsis mice (Figure 6D and Supplemental Figure 12G).

### Enhancement of the resolution of inflammation by VEGF-C156S depends on VEGFR-3/CCL21 pathway.

**Figure 7:**
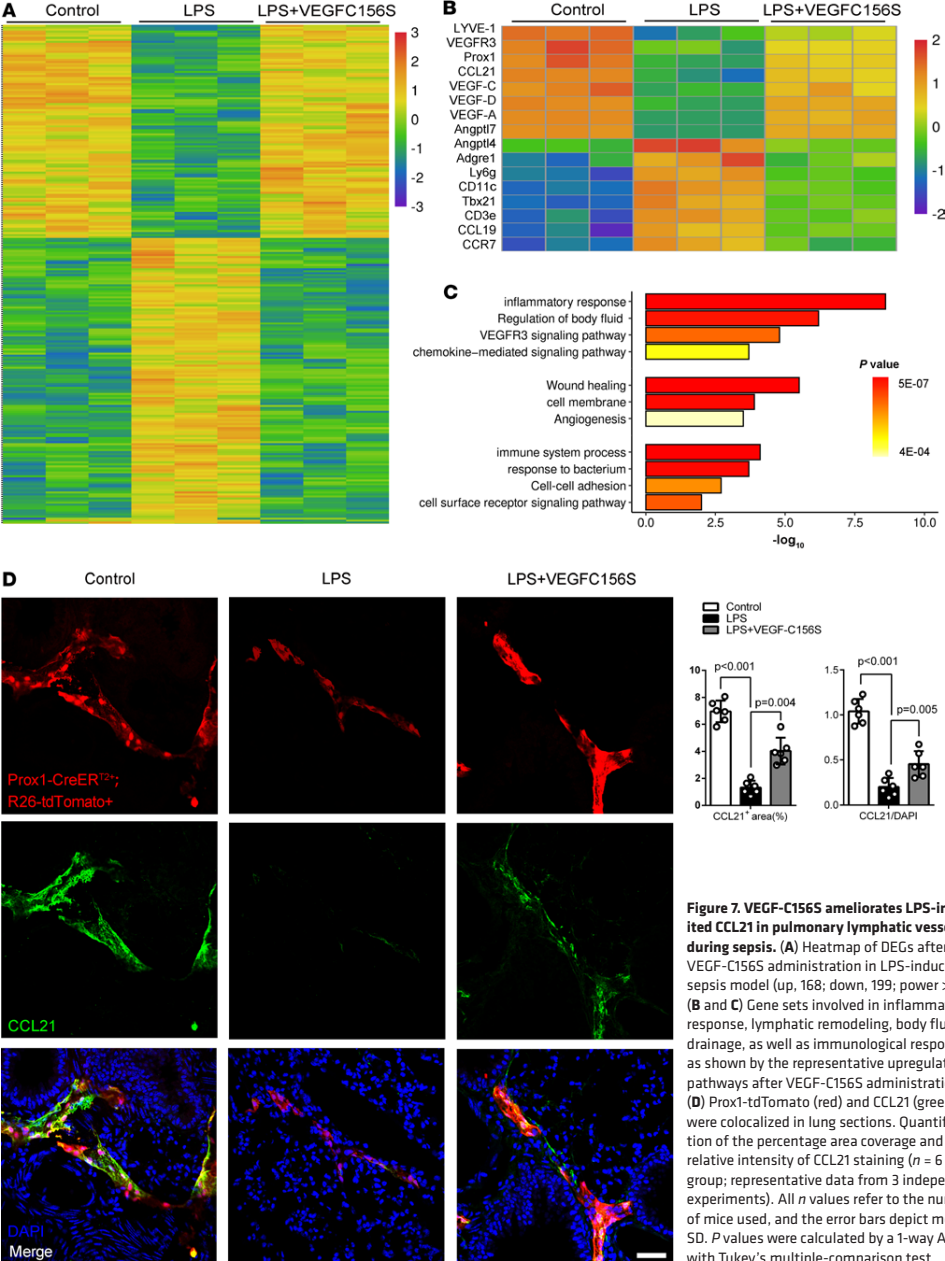
VEGF-C156S ameliorates LPS-inhibited CCL21 in pulmonary lymphatic vessels during sepsis. (**A**) Heatmap of DEGs after VEGF-C156S administration in LPS-induced sepsis model (up, 168; down, 199; power > 0.4). (**B** and **C**) Gene sets involved in inflammatory response, lymphatic remodeling, body fluid drainage, as well as immunological response as shown by the representative upregulated pathways after VEGF-C156S administration. (**D**) Prox1-tdTomato (red) and CCL21 (green) were colocalized in lung sections. Quantification of the percentage area coverage and the relative intensity of CCL21 staining (*n* = 6 per group; representative data from 3 independent experiments). All *n* values refer to the number of mice used, and the error bars depict mean ± SD. *P* values were calculated by a 1-way ANOVA with Tukey’s multiple-comparison test.

To better understand the mechanism of VEGF-C156S–mediated inflammation resolution, we analyzed the transcriptomic profile of the lung responding to VEGF-C156S treatment using RNA-Seq (Figure 7, A–C). We detected that several classical lymphatic markers such as CCL21 were markedly decreased in sepsis, while VEGF-C156S posttreatment could reverse those changes (Figure 7B). Meanwhile, those different genes also were also tested and verified by real-time PCR (data not shown). Notably, CCL21, produced and secreted specially by LECs, guides both recruitment and intraluminal directional crawling of CCR7^+^ lymphatic cells, such as macrophages and neutrophils, to migrate into the lymph node (23, 34, 35). We observed that CCL21 was colocalized with Prox1-tdTomato in pulmonary lymphatic vessels; moreover, the area coverage and the relative intensity of CCL21^+^ cells were significantly decreased in sepsis, while VEGF-C156S posttreatment could increase LPS-inhibited CCL21 expressions simultaneously (Figure 7D).

**Figure 8:**
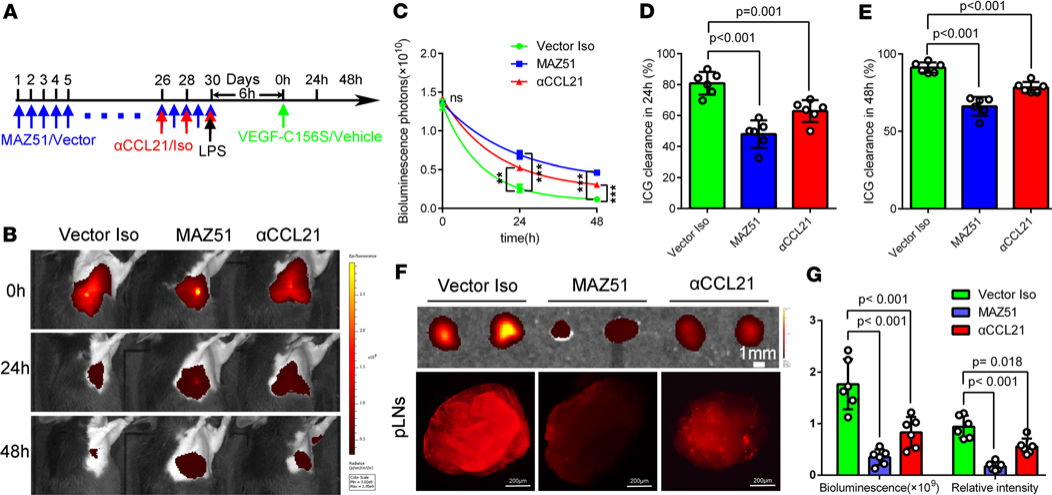
Enhancement of lymphatic drainage by VEGF-C156S is dependent on the VEGFR-3/CCL21 pathway. (**A**) Monitoring and treatment scheme. MAZ51 was intraperitoneally injected at 10 mg/kg of body weight for 30 days (5 days per week) for blocking VEGFR-3. CCL21 was blocked on days 2, 4, and 6 by CCL21-blocking antibody (αCCL21). VEGF-C156S was administrated to LPS-induced sepsis model following the administration of anti-CCL21 (αCCL21)/IgG (Iso) antibodies or MAZ51/Vector. (**B**–**E**) The lymphatic drainage function was determined by IVIS. The remaining ICG was quantified and presented as relative radiance at 0 hours, 24th hour, and 48th hour. The ICG clearance rate at the 24th hour and 48th hour (Vector Iso *n* = 6, MAZ51 *n* = 6; αCCL21 *n* = 6; representative data from 3 independent experiments). (**F** and **G**) Fluorescence intensities of ICG were determined in the pLNs at 30 minutes after the pour of ICG by IVIS and confocal microscopy at the 24th hour (Vector Iso *n* = 6, MAZ51 *n* = 6; αCCL21 *n* = 6; representative data from 3 independent experiments). All *n* values refer to the number of mice used, and the error bars depict mean ± SD. *P* values were calculated by a 1-way ANOVA with Tukey’s multiple-comparison test.

**Figure 9:**
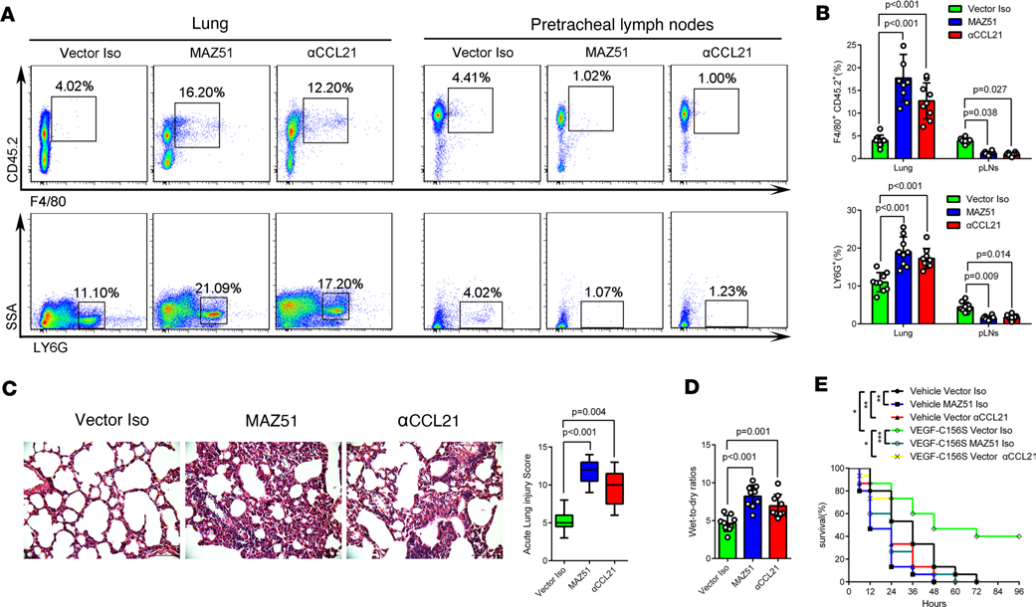
Enhancement of inflammation resolution by VEGF-C156S was dependent on VEGFR-3/CCL21 pathway. (**A** and **B**) Representative flow cytometry images and quantification of F4/80^+^/CD45.2^+^ cells and LY6G^+^ cells in lung and pLNs. (Vector Iso *n* = 9, MAZ51 *n* = 9; αCCL21 *n* = 9; representative data from 3 independent experiments). (**C**) Representative images of lung H&E-stained sections and the acute lung injury scores (Vector Iso *n* = 9, MAZ51 *n* = 9; αCCL21 *n* = 9; representative data from 3 independent experiments). The lung injury scores were shown as median (quartile) [M (P25, P75)] and analyzed by using the Kruskal-Wallis test. (**D**) Wet-to-dry ratios for lungs (Vector Iso *n* = 9, MAZ51 *n* = 9; αCCL21 *n* = 9; representative data from 3 independent experiments). Box plots show the interquartile range (box), median (line), and minimum and maximum (whiskers). All *n* values refer to the number of mice used, and the error bars depict mean ± SD. *P* values were calculated by a 1-way ANOVA with Tukey’s multiple-comparison test. (**E**) Survival of mouse with vehicle or VEGF-C156S treatment sepsis mice following the administration of anti-CCL21 (αCCL21)/IgG (Iso) antibodies, or MAZ51/Vector, were detected. Survival in each subgroup was estimated by Kaplan-Meier survival curves and compared by the pairwise log-rank test (*n* = 15 per group).

Next, we inhibited VEGFR-3 with MAZ51, a chemical inhibitor of VEGFR-3 with proven effectiveness in inhibiting lymphangiogenesis (22, 36), or blocked CCL21 with anti-CCL21 antibody (Figure 8A) (23). We found that the advantages of VEGF-C156S on pulmonary lymphatic drainage (Figure 8, B–G), the migration of macrophages and neutrophils (Figure 9, A and B), edema fluid clearance, and acute lung injury (Figure 9, C and D) were partly abolished by MAZ51 or anti-CCL21 antibody administration. In addition, MAZ51 or anti-CCL21 antibody also abolished the survival benefit of VEGF-C156S for sepsis mice (Figure 9E). Taken together, these results indicated that the enhanced effect of inflammation resolution by VEGF-C156S was dependent on the VEGFR-3/CCL21 pathway.

### Prior blocking of VEGFR-3/CCL21 also worsens sepsis-induced lymphatic dysfunction and inflammation.

We found that MAZ51 also reduced the expression of CCL21 (Supplemental Figure 13, A–F) and other lymphatic markers such as LYVE1 and Prox1 (Supplemental Figure 13H) but did not alter angiogenesis marker VEGFR-2 (Supplemental Figure 13G). Meanwhile, anti-CCL21 antibody did not affect the expression of VEGFR-3 and those lymphatic markers (Supplemental Figure 13, A–F and H). Meanwhile, blocking VEGFR-3 or CCL21 did not induce lung injury and tissue swelling in normal tissue (Supplemental Figure 13, I–K). Then, we found that prior blocking VEGFR-3 or CCL21 could reduce pulmonary lymphatic drainage (Supplemental Figure 14, A–G) and increase lung wet-to-dry ratios (Supplemental Figure 14H), pulmonary macrophage and neutrophil populations (Supplemental Figure 14I), acute lung injury scores (Supplemental Figure 14, J and K), and ear swelling (Supplemental Figure 14, L and M) in the sepsis mouse model, indicating that prior blocking of VEGFR-3/CCL21 could aggravate sepsis-induced lymphatic dysfunction and inflammation. In addition, prior blocking of VEGFR-3 or CCL21 augmented sepsis-induced mortality (Figure 9E).

## Discussion

Pulmonary inflammatory cells and edema fluid clearance are being recognized as significant medical issues for recovery from sepsis-induced ARDS (2, 8), yet the role of the pulmonary lymphatic vasculature remains poorly characterized. Here, our work provided evidence that pulmonary lymphatic structure was markedly impaired, resulting in lymphatic dysfunction. We further showed that posttreatment with VEGF-C156S restored lymphatic transport to reduce the pulmonary edema and the accumulation of the macrophages and neutrophils through the VEGFR-3/CCL21 pathway.

The lymphatic vasculature is mainly responsible for transporting immune cells and interstitial fluid into lymph nodes and back to the blood vessels finally during the tissue edema (19, 20, 37). The role of the pulmonary lymphatic vessels in the physiology and pathology of lungs has rarely been studied (27–31). Previous studies have attempted to determine the pulmonary lymphatic functions by pouring fluorescein-conjugated dextran intratracheally and then examining remaining pulmonary dextran discontinuously by fluorescence microscopy (27, 29, 30). Perhaps the difficulty of dynamically measuring the pulmonary lymphatic drainage function in vivo leads to the limitation of this research on the role of pulmonary lymphatic vessels on sepsis-induced ARDS, although several hypotheses have been proposed (2, 8, 38). Here, we improved this method by continuously examining residual pulmonary ICG in vivo using living imaging technology that measured the pulmonary lymphatic function in real time. Of note, high-dose ICG administration exacerbates lung injury and lengthens the time of clearance (39). Therefore, it was necessary to strictly control the dose of ICG.

Both acute- and chronic-phase inflammation produce different effects on the growth of lymphatic vessels. Chronic-phase inflammation in skin and pulmonary fibrosis show obvious local lymphangiogenesis, and LEC hyperplasia results from the crosstalk with VEGF-C secreted from macrophages (19, 20, 31). In the present work, we observed that LPS-induced sepsis mice had LEC-inhibiting effects on cell proliferation, migration, and tube formation. In addition, sepsis reduced the numbers of LECs and increased apoptotic LECs, indicating the dysfunction of LECs during the septic state. Thus, our results implied that sepsis-induced suppression of LECs’ proliferation and increase of LECs’ death could play important roles in lymphatic injury during sepsis.

We further showed that sepsis could lead to marked structural damage of pulmonary lymphatic vessels, shown as decreased lymphatic molecules and significant falloff and breakage of the lymphatic endothelium and the dysfunction of lymphatic barrier. Moreover, the prior deteriorated lymphatic dysfunction resulted in sepsis-induced pulmonary edema and inflammation, indicating that the impaired pulmonary lymphatic function by sepsis induced and deteriorated pulmonary edema and inflammation.

The clearance of pulmonary inflammatory cells and edema fluid is important for the recovery of sepsis-induced ARDS (2, 8, 32). The classical physiological studies proposed by Ernest Henry Starling held that pulmonary interstitial fluid is mainly reabsorbed by blood vessels in physiological conditions (27). However, the mechanism by which interstitial pulmonary edema fluid rich in proteins and inflammatory cells is cleared in ARDS remains unclear. In this work, we demonstrated that pulmonary lymphatic vessels are necessary for drainage of excessive pulmonary edema. Chronic inflammation such as lung fibrosis induces the expansion of pulmonary lymphatic vessels (40). Our results showed that pulmonary lymphatic vessels would expand and decrease subsequently in ARDS, a typical acute inflammatory state, indicating that acute or chronic inflammation would lead to different biological behaviors of lymphatic vessels.

VEGF-C has been reported to reduce inflammation in acute lung allograft rejection, bleomycin-induced pulmonary fibrosis, and other organ diseases (20, 31, 41). Meanwhile, VEGF-C/VEGFR-3 signaling restrains TLR4/NF-κB activation in macrophages to attenuate initial production of proinflammatory cytokines, indicating that VEGF-C itself has the effect of antiinflammation (42, 43). Antiinflammation focuses on blocking or antagonizing initiation steps in acute inflammation, while inflammation resolution is cessation of subsequent neutrophil influx and enhanced macrophage phagocytosis, microbial clearance, and efflux to lymph nodes (6, 7, 10). However, whether VEGF-C156S posttreatment can improve the resolution of inflammation in sepsis-induced ARDS has not been determined. In this work, we found that VEGF-C156S posttreatment could improve the sepsis-inhibited pulmonary lymphatic function to reduce pulmonary inflammation and edema fluid by boosting inflammatory cells draining to lymph nodes, indicating that ameliorated pulmonary lymphatic function by VEGF-C156S posttreatment could promote inflammation resolution. Moreover, the advantages of VEGF-C156S posttreatment on inflammation resolution and survival benefit could be abolished by blocking VEGFR-3, suggesting that the enhancement of the inflammation resolution by VEGF-C156S posttreatment depended on VEGFR-3.

To better understand the underlying mechanisms, we analyzed the transcriptomic profile and detected several lymphatic molecules, such as CCL21, which were markedly decreased, and VEGF-C156S posttreatment could reverse those changes. CCL21, produced and secreted specially by LECs, is required for inflammatory cells migrating into the lymph nodes (23, 34). We further showed that CCL21 was colocalized with Prox1 in pulmonary lymphatic vessels and was also reduced notably in sepsis-induced ARDS. Moreover, prior blocking of CCL21 worsened sepsis-induced accumulation of inflammatory cells, indicating that CCL21 played an important role in the migration of inflammatory cells. In addition, we determined that the enhanced effect of VEGF-C156S on the migration of inflammatory cells to lymph nodes could be abolished by blocking CCL21, demonstrating that the improvement of inflammation resolution by VEGF-C156S was dependent on the VEGFR-3/CCL21 pathway.

In summary, this work demonstrated that the pulmonary lymphatic drainage function was impaired because of the damage of lymphatic endothelium in sepsis-induced ARDS, and VEGF-C156S posttreatment improved pulmonary lymphatic drainage function to promote the resolution of inflammation through the VEGFR-3/CCL21 pathway. Therefore, our findings offer a therapeutic approach to sepsis-induced ARDS.

## Methods

Additional methods details are in Supplemental Methods.

### Animals.

Specific pathogen–free, C57BL/6J wild-type male mice (6–8 weeks old, 22–25 g) were purchased from Beijing Vital River Laboratory Animal Technology Co., Ltd. The *Prox1-CreER^T2^* line (C57BL/6) was a gift from Taija Makinen (Department of Immunology, Genetics and Pathology, Uppsala University, Uppsala, Sweden) (33). The *Prox1-CreER^T2^* mice were interbred with *R26-tdTomato* mice (C57BL/6, The Jackson Laboratory, catalog JAX: 007914) to generate *Prox1-CreERT2 Rosa26-tdTomato* mice. To induce Cre-mediated recombination, these mice were treated with 50 mg/kg tamoxifen (MilliporeSigma) in sunflower oil for 4 consecutive days by intraperitoneal injection. Four days after the last injection, the Cre recombination efficiency was confirmed (Figure 3A). The intensity of Prox1-tdTomato fluorescence was suitable for observing the lymphatic vessels 4 days after tamoxifen injection (Supplemental Figure 16). Mice were housed in groups of 4 per cage, maintained in a specific pathogen–free room with controlled habituation and temperature with regular 12-hour light/12-hour dark cycle, and fed with regular rodent chow and sterilized water.

### LPS-induced sepsis model.

To generate the LPS-induced sepsis mouse model, LPS (10 mg/kg) derived from *Escherichia coli* (MilliporeSigma) was intraperitoneally injected into male mice (44).

### CLP-induced sepsis model.

Male mice were subjected to middle-grade CLP using our previously described procedure with minor modification (44, 45). Experiments were performed under pathogen-free conditions, and the health status of mice was routinely examined by the raiser. Briefly, the mice were anesthetized with intraperitoneal injection of a mixture of ketamine (100 mg/kg) and xylazine (10 mg/kg) prior to all procedures. After anesthesia, the peritoneum was opened with a small midline abdominal incision in a sterile manner, and the cecum was exteriorized and ligated using 4-0 black silk distal to the ileocecal valve and through-and-through punctured with a 22 G needle. After puncturing, a small amount of stool was extruded through both punctures after the needle was removed to ensure intestinal patency. Then, the cecum was placed back into the abdominal cavity, and the abdominal incision was closed in 2 layers and daubed with lidocaine cream. Finally, 1 mL of prewarmed Ringer’s solution per 20 g body weight was injected subcutaneously. Sham-treated mice were undergoing exactly the same procedures except for the ligation and puncture of the cecum. Postoperative analgesia and volume supplement were repeated every 6–8 hours for 2 days. We evaluated the mice every 4 hours during the initial 48 hours after CLP.

### Treatment regimen.

At 6 hours after intraperitoneal injection of LPS or CLP (45–48), recombinant VEGF-C (Cys156Ser) protein (0.1 μg/g of body weight, R&D Systems) or PBS was randomly injected via tail vein (17, 18), and this time was the 0 hour (Figure 4A, Supplemental Figure 3A, and Supplemental Figure 8A). MAZ51 (catalog 676492, MilliporeSigma), a VEGFR-3 tyrosine kinase inhibitor, was dissolved in dimethyl sulfoxide and intraperitoneally injected for 30 days (10 mg/kg of body weight, 5 days per week). Meanwhile, the same volume of vector was given to the control group (22). CCL21 was blocked by anti-CCL21 antibodies (αCCL21, 2.5 μg/g of body weight, AF457, R&D Systems) 3 days, while the same volume of IgG (Iso) antibodies was given to the control group (23).

### Measuring the dermal lymphatic drainage function in vivo.

Lymphatic drainage function measurement was performed as previously described (19, 20). ICG-NIR lymphatic imaging was performed by injecting ICG (MilliporeSigma) into the local position and tracking the movement of ICG from the injection site to the draining lymph nodes. The dynamics of ICG fluorescence was visualized under infrared laser illumination and recorded by an IVIS (IVIS-Lumina-II, PerkinElmer). In brief, ICG (0.1 mg/mL, 6 μL, MilliporeSigma) was injected into the footpad or the ear using a graded Hamilton syringe (34-G needle) and moved from the injection site to the dpLNs or the deep cervical lymph nodes, respectively. Fluorescence intensities represented the amount of ICG determined at the 0 hour, 24th hour, and 48th hour using IVIS; were quantified; and were presented as relative radiance (photons/s per cm^2^/steradian) associated with the clearance rate to reflect the effect of tissue fluid clearance by lymphatic flow. At the 24th hour, ICG (0.1 mg/mL, 6 μL) was injected into the footpad or the ear, and then fluorescence intensities of ICG in lymph nodes (LNs) were determined by IVIS and confocal microscopy 30 minutes later. The speed of lymphatic flow was determined by assessing pulses of the ICG signal intensity in the lymphatic vessel within 10 minutes after footpad injection. Specifically, ICG was detected in the lymphatic vessel from 0 minutes to 10 minutes after injection and recorded every minute. The fluorescence intensity of ICG in the lymphatic vessel with every minute was recorded and quantified by IVIS. The speed of lymphatic flow = (10th minute ICG intensity – 0th minute ICG intensity)/10 min was determined as described previously (22).

### Lymphangiography.

Lymphangiography was performed with an Olympus multiphoton imaging system (FVMPE-RS) using a 25×/NA 1.05 water-dipping objective. The excitation wave length was tuned to 900 nm, and the emission filters were selected as 495–540 nm for the green channel. Briefly, the anesthetized animal was placed on a heated surface set at 37°C, and 70 kDa FITC-labeled dextran (5 mg/mL, 5 μL; MilliporeSigma) was injected subcutaneously into the footpad. Intravital imaging was performed on the skin of fossa iliaca by a tunable femtosecond pulse laser (MaiTai DeepSee, Spectra Physics) mounted on an upright microscope (BX63, Olympus). Lymphatic vessel flow was scored as unobstructed if a fluorescent pulse was visualized continuously in a vessel-like structure and at the left injected location (Supplemental Video 1). Conversely, lymphatic vessel flow was obstructed if a fluorescent pulse was discontinuous (Supplemental Video 2).

### Pulmonary lymphatic drainage function measurement in vivo.

We improved a previous method to measure the pulmonary lymphatic drainage function by ICG-NIR lymphatic living imaging technology in vivo. We observed that low-dose ICG (1 mg/mL, 10 μL) did not cause or deteriorate lung injury during normal or septic state (Supplemental Figure 17). Then, ICG (1 mg/mL, 10 μL) was intratracheally poured into unilateral lung by tracheal intubation with fixed cannula depth. Then the lungs were dissected, and the pulmonary lymphatic vessels were stained while the blood vessels were not stained in normal or sepsis mice. Moreover, the pLNs were also stained (Supplemental Figure 1). Multiphoton imaging showed that the ICG drained primarily via the lung lymphatic vessels rather than blood vessels in normal or sepsis mice (Supplemental Videos 3 and 4). The remains of ICG in lung were determined at the 0 hour, 24th hour, and 48th hour using IVIS, which was associated with the clearance rate to reflect the effect of pulmonary fluid clearance by lymphatic drainage (Figure 1B).

### Immunofluorescence.

For immunofluorescence, the lung tissue and pLNs were fixed in 10% neutral buffered formalin, then embedded in paraffin wax and cut into 5 μm sections. The deparaffinized sections were subjected to antigen retrieval, blocked by 10% normal goat serum with 0.5% PBS-Tween (PBST) for 1 hour at room temperature, and stained overnight with primary antibodies at 4°C. After rinsing with PBST 3 times for 10 minutes each, secondary antibodies were incubated for 1 hour at room temperature. Immunofluorescence staining on sections was performed using primary antibodies against Lyve-1 (rabbit, 1:500; Abcam, catalog ab14917), VEGFR-3 (goat, 1:500, R&D Systems, AF743), VE-cadherin (rabbit, 1:500; Abcam, catalog ab205336), CCL21 (rabbit, 1:500; Abcam, catalog ab231116), F4/80 (rabbit, 1:100; Abcam, catalog ab16911), LY6G (rat, 1:100; Abcam, catalog ab210204), CD68 (rat, 1:200; Abcam, catalog ab237968), CD31 (rabbit, 1:100; Abcam, catalog ab222783), and Ki-67 (rabbit, 1:200; Thermo Fisher Scientific, catalog PA5-19462), or TUNEL-FITC (Abcam, catalog ab66108). The corresponding secondary antibodies were used: goat anti-rabbit DyLight 594 (1:200, Invitrogen, catalog 35560), goat anti-rabbit Alexa Fluor 488 (1:200, Abcam, catalog ab150077), donkey anti-goat Alexa Fluor 488 (1:200, Invitrogen, catalog A32814), donkey anti-goat Alexa Fluor 594 (1:200, Abcam, catalog ab 150132), and goat anti-rat Alexa Fluor 488 (1:500, Cell Signaling Technology, catalog 4416S/4417S).

### Image analysis.

Images were acquired with a Nikon C2si confocal microscope with a resolution of 2,048 × 2,048 pixels and a *z*-step of 2,600 μm. The exposure time and brightness/contrast of each image were applied equally for all images, which were analyzed using the ImageJ software (NIH). The mean value of 5 sections of each lung or pLNs was used for each animal. The area of lymphatic vessel per field was calculated, and 4 to 5 fields of each sections were quantified to acquire the mean value. Prox1 did not only label LECs, so VEGFR-3 and CD31 were also used simultaneously to label LECs. The percentage of pulmonary lymphatic vessels was defined by dividing the area of anti–VEGFR-3/anti–LYVE1/anti-CD31/Prox1-tdTomato labeled by the area of the lung section or the ear section. The percentage of macrophages was defined by dividing the area of anti-F4/80/anti-CD68 antibody labeled by the area of the lung section. The percentage of neutrophils was defined by dividing the area of anti-LY6G antibody labeled by the area of the lung section. The percentage of pLNs’ lymphatic vessels was determined by dividing the area of anti–VEGFR-3 antibody labeled per section. The percentage of macrophages/neutrophils was defined by dividing the area of anti-F4/80/anti-LY6G antibody labeled by the area of the pLN section. Five sections of each pLN were quantified to acquire the mean value. Raw data were collected using Microsoft Excel 2007 software, and the group allocation was blinded.

### Flow cytometry.

Mouse lung was dissected after transcardial perfusion by cold PBS, then minced into small pieces. The LN was teased apart into single-cell suspensions by pressing with the plunger of a 5 mL syringe. Lung and LN tissues were digested by collagenase I (0.5 mg/mL, MilliporeSigma, catalog 9001-12-1) at 37°C for 30 minutes to yield the dispersed cells, then filtered by 60 μm nylon mesh cell strainers to eliminate clumps and debris. RBCs were eliminated using RBC lysis buffer. Cells were stained for live cells by Fixable Viability Dye eFluor 506/780 (Invitrogen, catalog 65-2860-40) and the following antibodies: mouse anti-CD45.2 FITC-conjugated antibody (11-0454-82, eBioscience), rat anti-F4/80 allophycocyanin-conjugated antibody (17-4801-82, eBioscience), rat anti-Ly6G PerCP-Cy5.5–conjugated antibody (35-5931-82, eBioscience), rat anti-CD31 PE-Cyanine7–conjugated antibody (25-0311-82, eBioscience), Syrian hamster anti-Podoplanin PE-conjugated antibody (12-5381-82, eBioscience), and Annexin V FITC conjugate (A13199, eBioscience). Flow cytometry was performed using BD FACSAria II and analyzed by the FlowJo 7 software, and investigators were blinded to the group allocations.

### RNA-Seq analysis.

RNA-Seq analysis was performed as previously described (23). Three independent RNA samples per group from the control group, LPS group, and LPS+VEGF-C group were subjected to RNA-Seq. In brief, the lymphatic vessels, located near the bronchus, were stained light green after the ICG pulmonary perfusion (Supplemental Figure 1). Then, these lymphatic vessels were isolated under the dissecting microscope for further RNA-Seq. FACS showed that the LECs’ (CD45^–^, Prox1^+^, CD31^+^) purity in isolated lymphatic vessels was more than 85%. Total RNA was extracted from lymphatic vessels and isolated with TRIzol (Invitrogen, A33250) according to the manufacturer’s instructions for microarray analysis. Samples were subjected to sequencing on an Illumina HiSeq 4000 (LC Sciences) following the manufacturer’s recommended protocol. Based on KEGG Mapper (https://www.kegg.jp/kegg/tool/map_pathway2.html), the fold-changes were analyzed by filtering the data set with *P* ≤ 0.05 and power ≥ 0.4 for screening out differentially expressed genes (DEGs). Gene Ontology analysis and heatmaps of DEGs and enriched gene sets were created with OmicStudio tools (https://www.omicstudio.cn/tool).

### Statistics.

The data were presented as the mean ± SD, with differences between mean values determined by 2-tailed paired or unpaired Student’s *t* test, 1-way ANOVA, or 1-way ANOVA with Tukey’s multiple-comparison test using Prism 7.0 software (GraphPad Software) and SPSS 16.0 software (IBM Corp.). Survival rate in each subgroup was estimated by Kaplan-Meier survival curves and compared by the pairwise log-rank test. Only the lung injury scores were categorical variables, and abnormal distribution by Kolmogorov-Smirnov test was shown as median (quartile) [M (P25, P75)]. Differences of the lung injury scores were analyzed by using the Kruskal-Wallis test; multiple comparisons were derived from the Mann-Whitney *U* test. There were no missing data. *P* values less than 0.05 were considered statistically significant. During analysis blinding was applied to the group allocations.

### Study approval.

All animal procedures were approved by the Laboratory Animal Ethics Committee of Wenzhou Medical University (WMU-2018-064), and animal experiments were performed according to the Laboratory animal-Guideline for ethical review of animal welfare (GB/T 35892-2018, China).

### Data availability.

This study includes no data deposited in external repositories. All data generated or analyzed during this study are included in the article and its Supporting Data Values XLS file.

## Author contributions

SWJ, YH, FGS, and PHZ conceived the project and designed experiments; PHZ, CHW, and WWZ conceived the experiments and analyzed data; PHZ, SSW, YDD, XYW, CHW, and WWZ performed experiments; PHZ and SSW conducted the mouse experiments; PHZ and CHW performed the lymphatic imaging experiments; WWZ and XYW performed the flow cytometry and interpreted these data; WWZ and YDD performed the cellular experiments; PHZ interpreted the data and wrote the manuscript; and SWJ and YH assisted in data interpretation and edited the manuscript. All authors have approved the final version of the manuscript and have agreed to be accountable for all aspects of the work.

## Supplementary Material

Supplemental data

Supplemental video 1

Supplemental video 2

Supplemental video 3

Supplemental video 4

Supporting data values

## Version 1 11/16/2023

In-Press Preview

## Version 2 01/09/2024

Electronic publication
