# Supplementary material for: Efficient pulmonary lymphatic drainage is necessary for inflammation resolution in ARDS
Source: JCI Insight. 2024 Jan 9;9(1):e173440. doi: 10.1172/jci.insight.173440 (PMC10906459; doi:10.1172/jci.insight.173440)
Supplement: Supplemental data [file jciinsight-9-173440-s067.pdf]

Caption for supplementary material

Supplemental Methods

1. Western blotting
2. RT-qPCR
3. Evans Blue exudation assay
4. Scanning electron microscope
5. Pathological and biological studies

Supplemental Figures

Supplemental Figure 1: Detecting the pulmonary lymphatic drainage by ICG imaging.

Supplemental Figure 2: The dermal lymphatic drainage function was impaired in LPS-induced sepsis.

Supplemental Figure 3: Sepsis caused dysfunction of blood endothelial barrier.

Supplemental Figure 4: The pulmonary lymphatic vessels were damaged in CLP-induced sepsis model.

Supplemental Figure 5: VEGF-C156S post-treatment rejuvenated the lymphatic vessels through reducing LECs death and promoting proliferation.

Supplemental Figure 6: Sepsis caused the impairments of dermal lymphatic vessels.

Supplemental Figure 7. LPS-induced sepsis mice had severe pulmonary edema and inflammatory response.

Supplemental Figure 8: Post-treatment of VEGF-C156 ameliorated pulmonary lymphatic drainage function through rejuvenating lymphatics in CLP-induced sepsis.

Supplemental Figure 9: Post-treatment of VEGF-C156S reduced the sepsis-induced edema through repairing the lymphatic vessel.

Supplemental Figure 10: Post-treatment of VEGF-C156S ameliorated dermal lymphatic drainage function in LPS-induced sepsis.

Supplemental Figure 11: VEGF-C156S post-treatment reduced the accumulation of pulmonary macrophages through lymphatic drainage during sepsis.

Supplemental Figure 12: VEGF-C156S post-treatment promoted pulmonary

inflammatory cells to drain to pLNs in CLP-induced sepsis.

Supplemental Figure 13: Effect of the VEGFR3 inhibitor (MAZ51) or anti-CCL21 antibodies (αCCL21) on pulmonary lymphangiogenesis.

Supplemental Figure 14: Blocking VEGFR3 or CCL21 deteriorated sepsis-induced lymphatic dysfunction and inflammation.

Supplemental Figure 15. The area of pulmonary lymphatic vessels was significantly reduced in sepsis mice.

Supplemental Figure 16. Observing the lymphatic vessels by PROX1-tdTomato fluorescence.

Supplemental Figure 17. Low-dose ICG does not cause or deteriorate lung injury during normal or septic state.

Supplemental Figure 18. VEGF-C156S ameliorated pulmonary lymphatic drainage function in sepsis-induced ARDS by using FITC-dextran IVIS.

Supplemental Figure 19. VEGF-C156S ameliorates pulmonary lymphatic integrity in sepsis.

Supplemental Figure 20. Identification of dead LECs by flow cytometry.

Supplementary Videos

Supplementary Video 1. The consecutive lymphatic flow in control mice.

Supplementary Video 2. The obstructed lymphatic flow in sepsis mice.

Supplementary Video 3,4. ICG drains primarily via the pulmonary lymphatic vessels in control or sepsis mice.

Supplementary Table

Supplementary Table 1. The sequences of the primers used.

Supplemental Methods

1. Western blotting

For western blots, lung tissues were lysed in chilled RIPA buffer containing protease inhibitor and phosphatase inhibitor for 30 minutes on ice. 10 μg protein was loaded for SDS-PAGE with standard western blotting procedures. The following primary antibodies and at a 1:1000 dilution: Anti-LYVE-1 antibodies (Abcam, Cat. No. ab183501), Anti-VEGFR3 antibodies (R&D System, Cat. No. AF743) and Anti-CCL21 antibodies (R&D System, Cat. No. AF457). The following secondary antibodies were used: donkey anti-rabbit IgG (HRP) and donkey anti-goat IgG (HRP). The proteins were detected using enhanced chemiluminescence (all GE Healthcare) and processed using Quantity One.

2. RT-qPCR

Total RNA was extracted with TRIzol Reagent (Invitrogen, Cat. No. A33250). cDNAs were synthesized with the PrimeScript RT Master Mix (Takara, RR036B) and subjected to RT-qPCR using specific primers. The sequences of the forward and reverse primers are shown in Supplementary Table 1.

3. Evans Blue exudation assay

Lymphatic barrier function was visualized in the Miles assay by exudation of Evans blue as previous reported (1). Briefly, mice were anesthetized and then 20 μL Evans Blue (1% in PBS) was injected into the footpad and left circulating for 30 minutes. Subsequently, skin patches (10mm×5mm) close to the injection sites were incubated in formamide for 24 hours. Extracted Evans Blue and hemoglobin was measured spectrophotometrically at 610 and 450 nm, respectively. The Evans Blue/hemoglobin ratio is given.

4. Scanning electron microscope

For scanning electron microscope, ICG (green) was intratracheally poured into the lung by tracheal intubation, and then the lymphatic vessels which located near

bronchus were stained into green own to the lymphatic drainage (Figure S1A). Subsequently, as previous described (2), these lymphatic vessels were isolated under the dissecting microscope and then cut into slices of 5 x 5 mm^2, followed by fixation in 4% PFA and 2.5% glutaraldehyde in 0.1 M sodium cacodylate buffer for 2 hours at room temperature. Samples were incubated in 1% osmic acid for 1 hour, submerged in 2% tannic acid for 2 hours for electrical conduction, and progressively dehydration in increasing ethanol concentrations, followed by rinsing with isoamyl acetate. Then the specimens were dried in an Eiko HCP-2 Critical Point Dryer at 1,200 psi and 32°C for 1h, and sputter coated with gold by an Eiko IB-5 ionic sprayer meter. The specimens were examined at an accelerating voltage of 20kV using a Stereoscan 260 Hitachi SEM (Japan).

5. Pathological and biological studies

At 24th hour after sepsis mice formation or the recombinant VEGF-C156S protein treatment, the mice were anesthetized with ketamine (100 mg/kg) and xylazine (10 mg/kg). Then, anesthetized mice were exsanguinated, and the lung, ear and pLNs tissue samples were immediately obtained. The samples were embedded in paraffin, stained with hematoxylin and eosin (H&E), and analyzed by using a light microscope (OLYMPUS,BX53F). Organ injury scores were determined by two independent investigators blinded to the research assignment and based on alveolar congestion, alveolar haemorrhage, alveolar wall/hyaline hyaline membrane thickness and inflammatory cell accumulation. In brief, 0 represented no injury, 1 represented slight injury (25%), 2 represented moderate injury (50%), 3 represented severe injury (75%), 4 represented very severe injury (almost 100%). The sum of the four variables represented the lung injury score (total score: 0-16)(3). For the ears, the swelling of skin scored by the thickness of ears, as inflammatory edema causes an increase in tissue. The lung tissue samples were homogenized with phosphate buffer to obtain the supernatant liquid for detecting the inflammatory factors. Briefly, the concentrations of MPO, IL-1β, IL-6, TNF-α in lung tissue homogenate and the serum were determined by using ELISA kits in accordance with the R&D system's protocols.

References

1. Aman J, van Bezu J, Damanafshan A, Huveneers S, Eringa EC, Vogel SM, Groeneveld AB, Vonk Noordegraaf A, van Hinsbergh VW, and van Nieuw Amerongen GP. Effective treatment of edema and endothelial barrier dysfunction with imatinib. *Circulation.* 2012;126(23):2728-38.

2. Bazigou E, Lyons O, Smith A, Venn G, Cope C, Brown N, and Makinen T. Genes regulating lymphangiogenesis control venous valve formation and maintenance in mice. *The Journal of clinical investigation.* 2011;121(8):2984-92.

3. Sartori C, and Matthay M. Alveolar epithelial fluid transport in acute lung injury: new insights. *The European respiratory journal.* 2002;20(5):1299-313.

Supplemental figure and legends

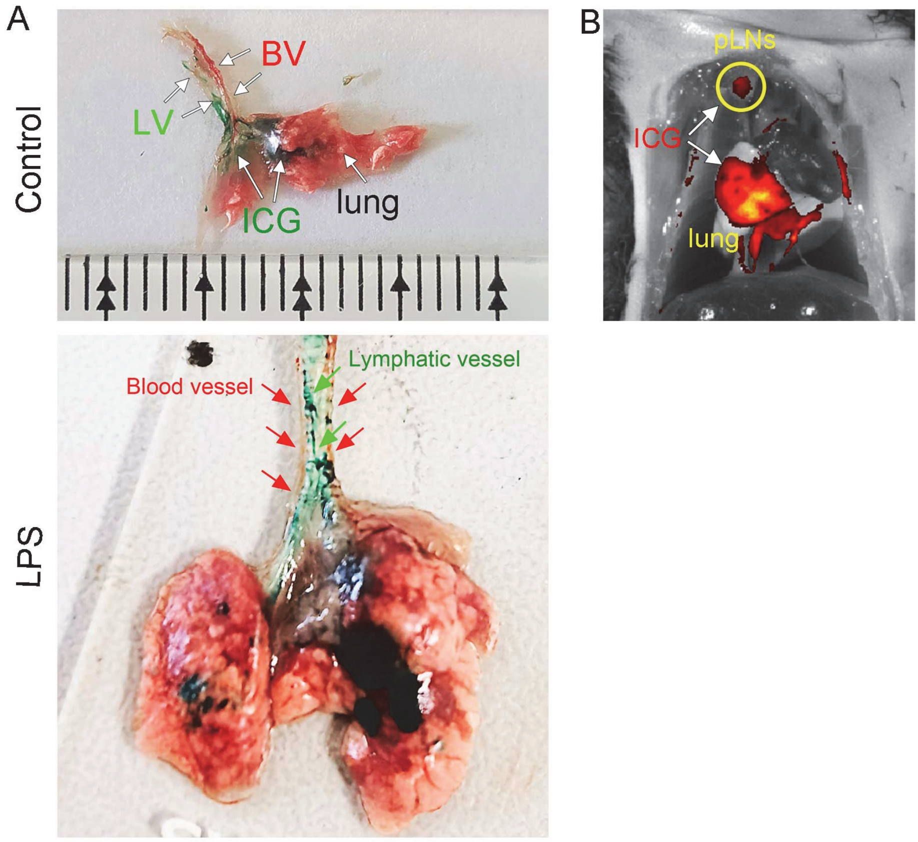

Supplemental Figure 1: Detecting the pulmonary lymphatic drainage by ICG imaging.

(a) Indocyanine Green (ICG, 1mg/mL, 10μL, green), a near-infrared (NIR) fluorescent dye, was intratracheally poured into the lung by tracheal intubation. 30 min later, isolating the lung. LV: lymphatic vessel, BV: blood vessel. **(b)** ICG drained into the pretracheal lymph nodes (pLNs) from the lung.

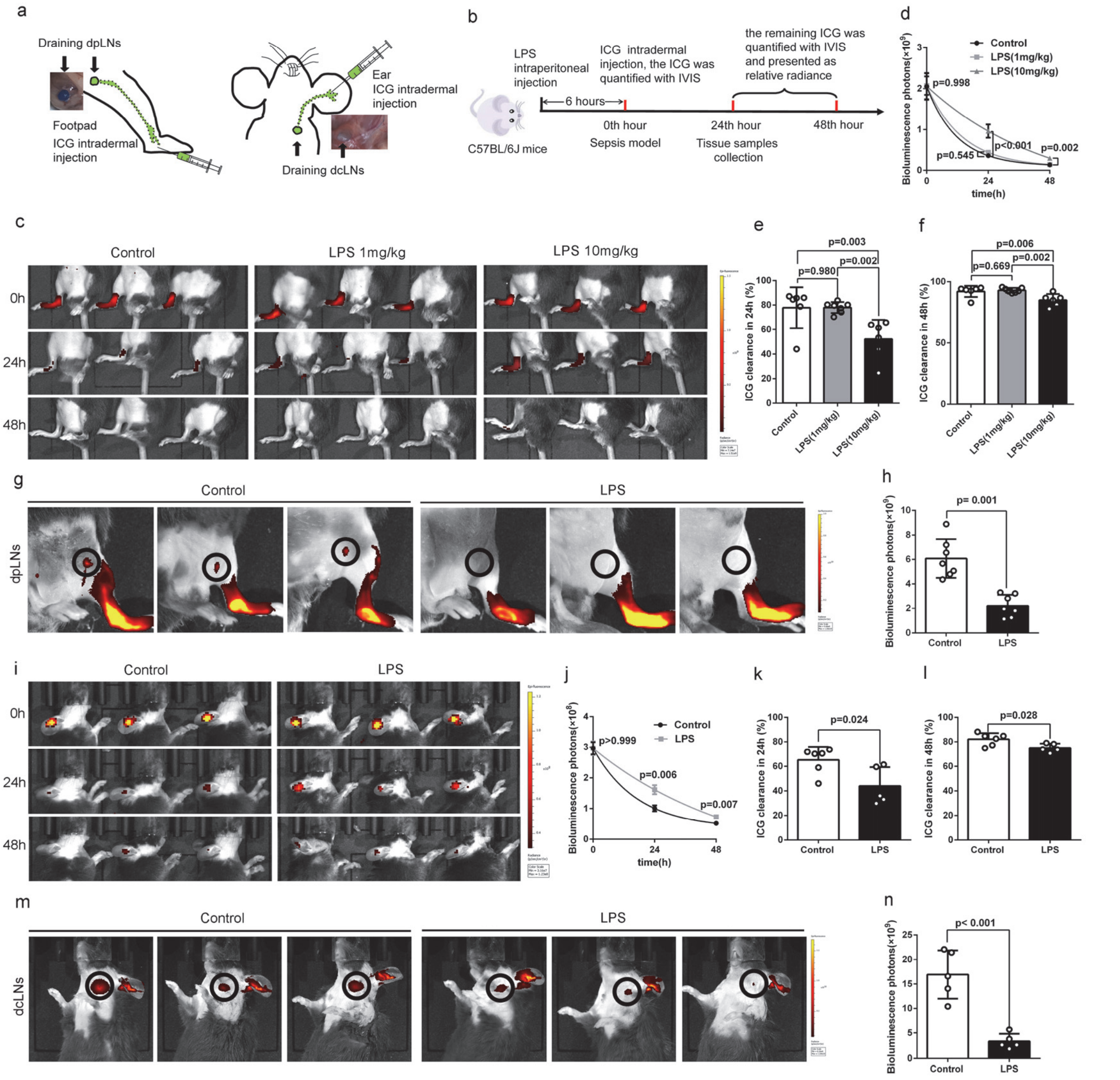

Supplemental Figure 2: The dermal lymphatic drainage function was impaired in LPS-induced sepsis.

(a) Indocyanine Green (ICG) was injected intradermally into the footpad or the ear, and the movement of ICG from the injection site to the draining popliteal lymph nodes (dpLNs) or the deep cervical lymph nodes (dcLNs). **(b)** Procedure and timeline: At 6 hours after the intraperitoneal injection of LPS (10mg/kg), the sepsis model was induced. Meanwhile, ICG (0.1mg/mL, 6 μL) was intradermally injected into the skin. Fluorescence intensities were determined at 0th hour, 24th hour and 48th hour using IVIS associated with the clearance rate to reflect the effect of tissue fluid clearance by lymphatic flow. The tissue samples were collected at 24th hour. **(c-f)** ICG was intradermally injected into the footpad of different dose LPS-induced sepsis mice. The remaining ICG were determined at 0th hour, 24th hour and 48th hour using IVIS,

quantified, and presented as relative radiance (photons/sec per cm2/steradian). Panel c right bar: the color scale indicates fluorescence intensity. Upper yellow color indicates maximum fluorescence intensity, whereas lower red color indicates minimum fluorescence intensity. The ICG clearance rate at 24th hour and 48th hour (n=6 to 7; representative data from three independent experiments). **(g-h)** At 24th hour, ICG (0.1mg/mL, 6 μL) was intradermally injected into the footpad. Fluorescence intensities were determined in dpLNs at 30 minutes after the injection, quantified, and presented as relative radiance (Control n=7, LPS n=7; representative data from three independent experiments). **(i-l)** ICG (0.1mg/mL, 6 μL) was intradermally injected into the skin of ear and quantified by IVIS. The ICG clearance rate at 24th hour and 48th hour (Control n=6, LPS n=5; representative data from three independent experiments). **(m-n)** At 24th hour, ICG was intradermally injected into the skin of ear. Fluorescence intensities were determined in dcLNs at 30 minutes after the injection (Control n=5, LPS n=5; representative data from three independent experiments). p values were calculated by a one-way ANOVA with Tukey's multiple comparison test or two-tailed paired or unpaired Student's t-test.

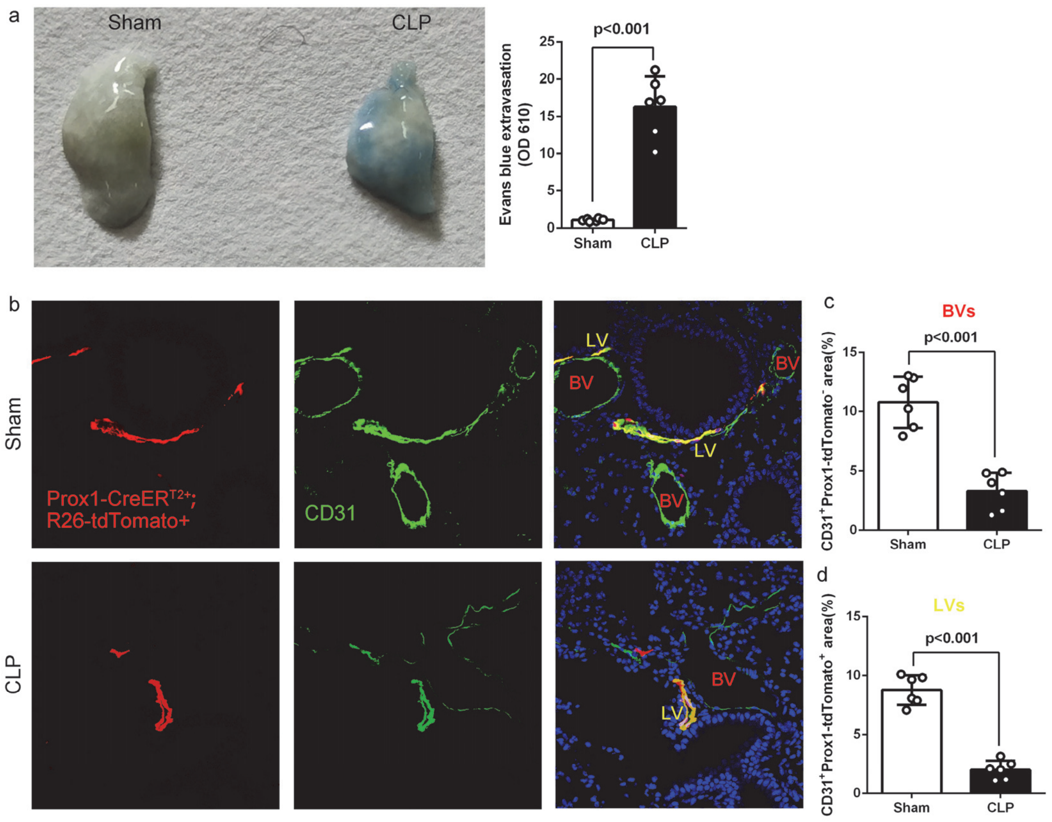

Supplemental Figure 3: Sepsis caused dysfunction of blood endothelial barrier. **(a)** The pulmonary blood barrier was assessed by Evans Blue exudation. 20 μL Evans Blue (1% in PBS) was injected from the tail vein and left circulating for 30 minutes. Subsequently, the lungs were extracted after the perfusion. Quantification of formamide-extracted Evans Blue (610 nm) by spectrophotometer. **(b-d)** Pulmonary lymphatic vessels (LVs) were labeled with $CD31^{+}$Prox1-$tdTomato^{+}$ area, while blood vessels (BVs) were labeled with $CD31^{+}$Prox1-$tdTomato^{-}$ area (Sham =6, CLP=6; representative data from three independent experiments). All n values refer to the number of mice used and the errors bars depict mean ± SD. p values were calculated by two-tailed paired or unpaired Student's t-test.

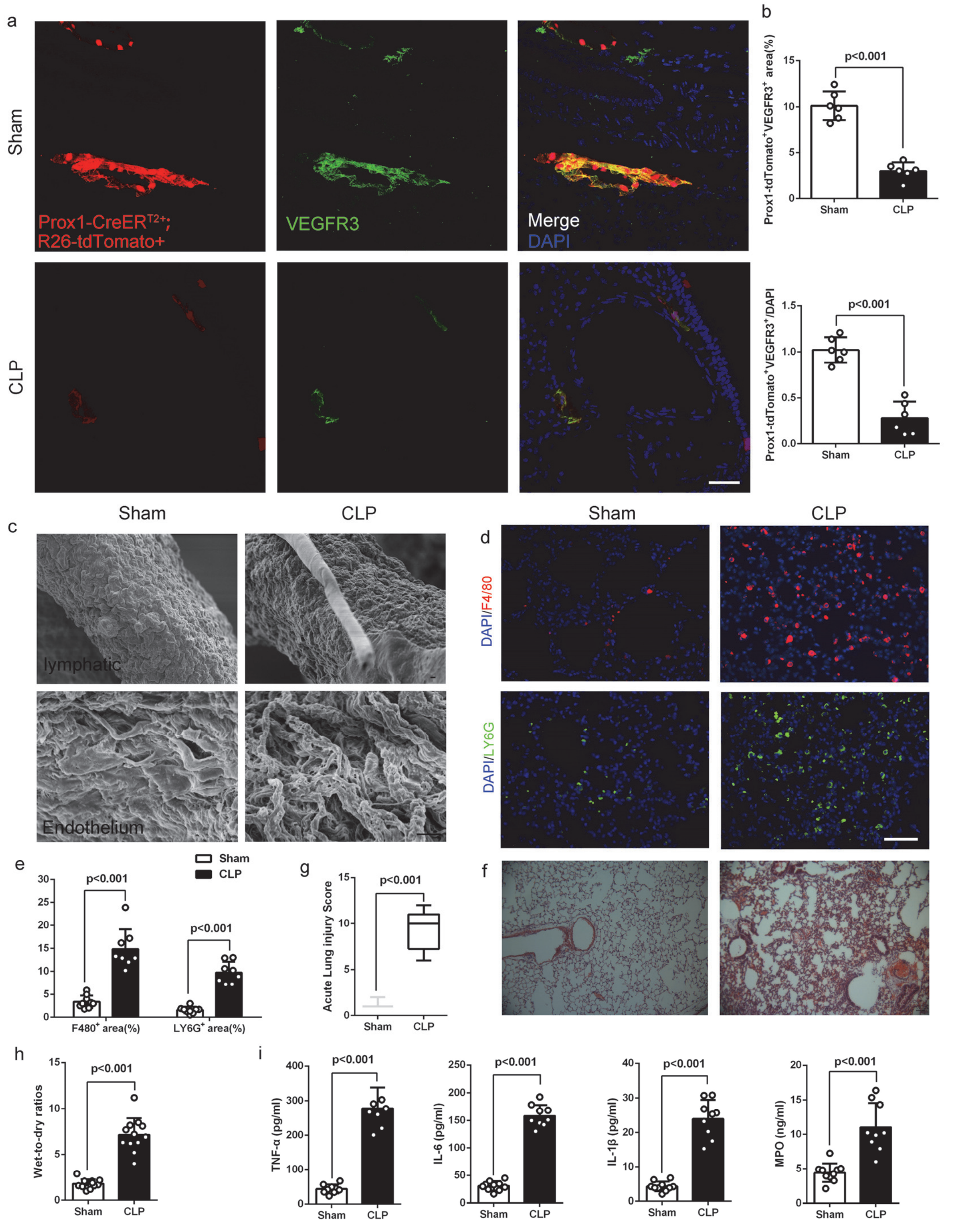

Supplemental Figure 4: The pulmonary lymphatic vessels were damaged in CLP-induced sepsis model.

At 24th hour, the lung tissue was harvested. **(a)** Lymphatic vessels were labeled with immunofluorescence stain of VEGFR3 (green) and Prox1-tdTomato (red) signals from *Prox1-CreERT2+;R26-tdTomato+* mice after tamoxifen induction. Scale bars, 50 μm. **(b)** Quantification of the percent area coverage and the relative fluorescence intensity of lymphatic vessels (Sham n=6, CLP n=6; representative data from three independent

experiments). **(c)** Scanning electron microscopy of the pulmonary lymphatic vessels and the lymphatic endothelium. The lung tissue was frozen and then cut into slices of 5 x 5 mm^2 for Scanning electron microscopy study (representative images from two independent experiments). Scale bars, 1μm. **(d,e)** Representative images and quantification of F4/80$^+$ cells (macrophages) and LY6G$^+$ cells (neutrophils) in lung sections. Scale bars, 50 μm. (Sham n = 9, CLP n = 8; representative data from three independent experiments). **(f,g)** Representative images of lung H&E-stained sections and the acute lung injury scores were determined by two independent investigators following the applicable criteria. Scale bars, 50 μm. (Sham n = 12, CLP n = 12; representative data from three independent experiments). **(h)** Wet-to-dry ratios for lungs (Sham n = 12, CLP n = 12; representative data from three independent experiments)**. (i)** The concentrations of inflammatory factors in lung tissue homogenate, such as TNF-α, MPO, IL-1β, and IL-6, were measured by ELISA (Sham n = 10, CLP n = 9; representative data from three independent experiments). All n values refer to the number of mice used and the error bars depict mean±SD. p values were calculated by two-tailed unpaired Student's t-test. Only the lung injury scores were shown as Median (Quartile) [M (P25, P75)] and analyzed by using the Kruskal-Wallis test.

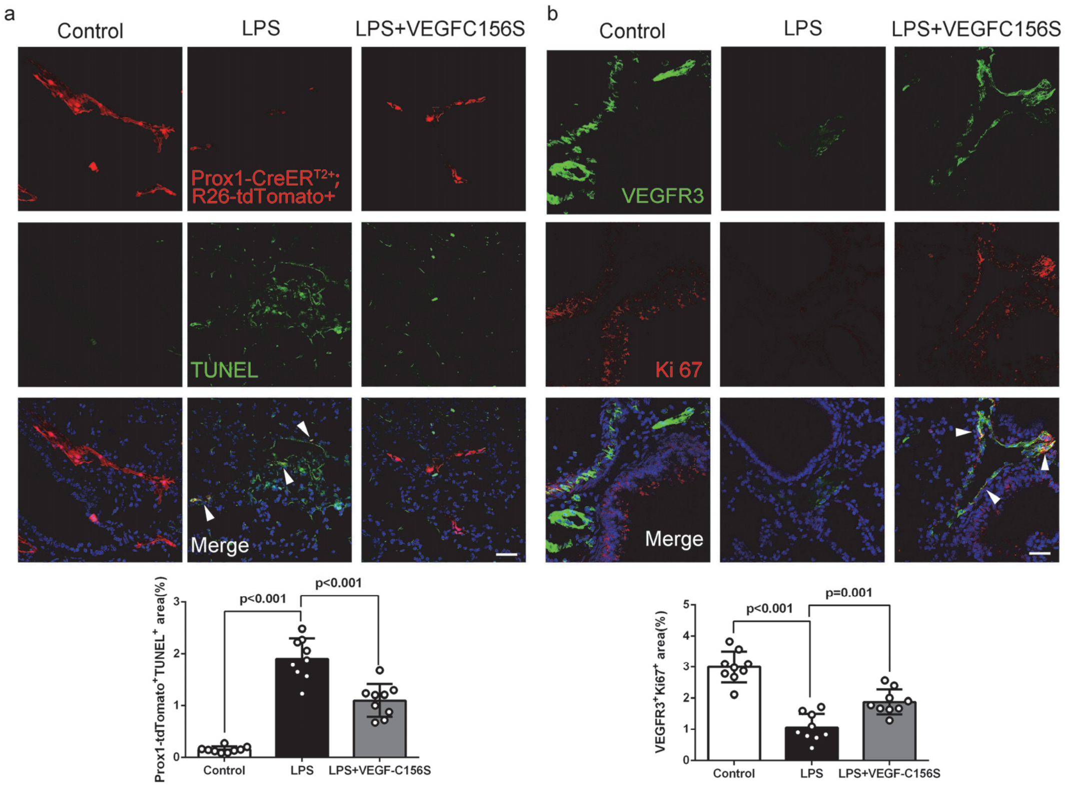

Supplemental Figure 5: VEGF-C156S post-treatment rejuvenated the lymphatic vessels through reducing LECs death and promoting proliferation.

(a) Pulmonary lymphatic vessels (LVs) were costained with cell dead marker TUNEL. Scale bar, 50 μm. **(b)** LVs were costained with proliferative marker Ki67. Scale bar, 50 μm. All n values refer to the number of mice used and the error bars depict mean±SD. p values were calculated by a one-way ANOVA with Tukey's multiple comparison test.

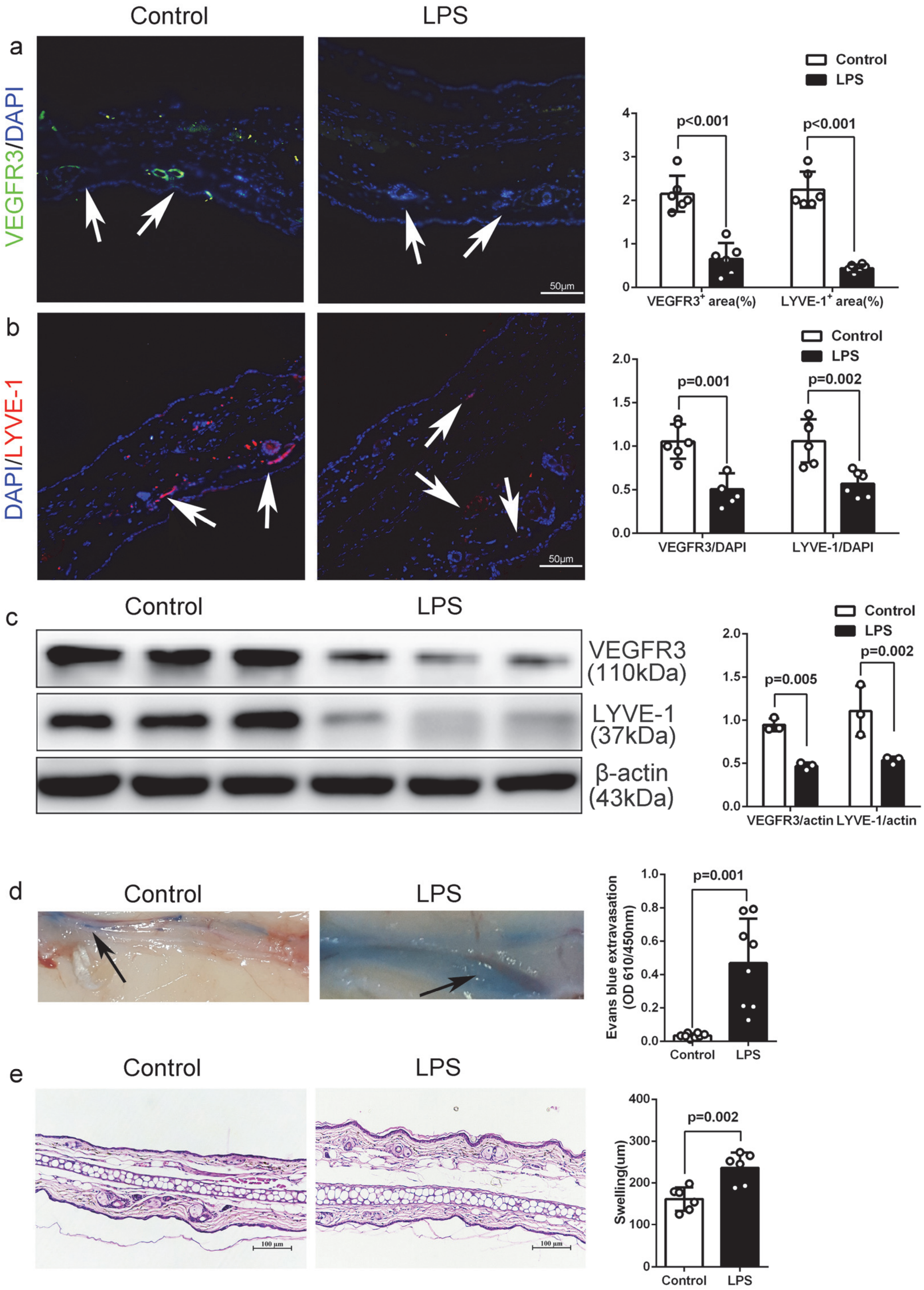

Supplemental Figure 6: Sepsis caused the impairments of dermal lymphatic vessels.

At 24th hour, ears were obtained from LPS-induced sepsis mice. **(a-b)** Ear sections were subjected to immunofluorescence stain for VEGFR3 (green) and LYVE-1 (red) for lymphatic vessels. Representative images of dermal lymphatic vessels and quantification of the percent area coverage and the relative intensity of VEGFR3 or

LYVE-1 staining (Control =6, LPS=6; representative data from three independent experiments). **(c)** VEGFR3 and LYVE-1 protein levels in dermal tissue were detected by western blot (Control =3, LPS=3; representative data from three independent experiments). **(d)** 20 μL Evans Blue (1% in PBS) was injected into the footpad and left circulating for 30 minutes. Subsequently, the foreleg skin (10mm×5mm) close to the injection sites was incubated in formamide for 24 hours. Quantification of formamide-extracted Evans Blue (610 nm) by spectrophotometer and corrected for hemoglobin (450 nm, Control =8, LPS=8; representative data from three independent experiments). **(e)** Representative images of ear skin H&E-stained sections. The severities of swelling were scored by the thickness of ears (Control =6, LPS=6; representative data from three independent experiments). All n values refer to the number of mice used and the errors bars depict mean ± SD. p values were calculated by two-tailed unpaired Student's t-test.

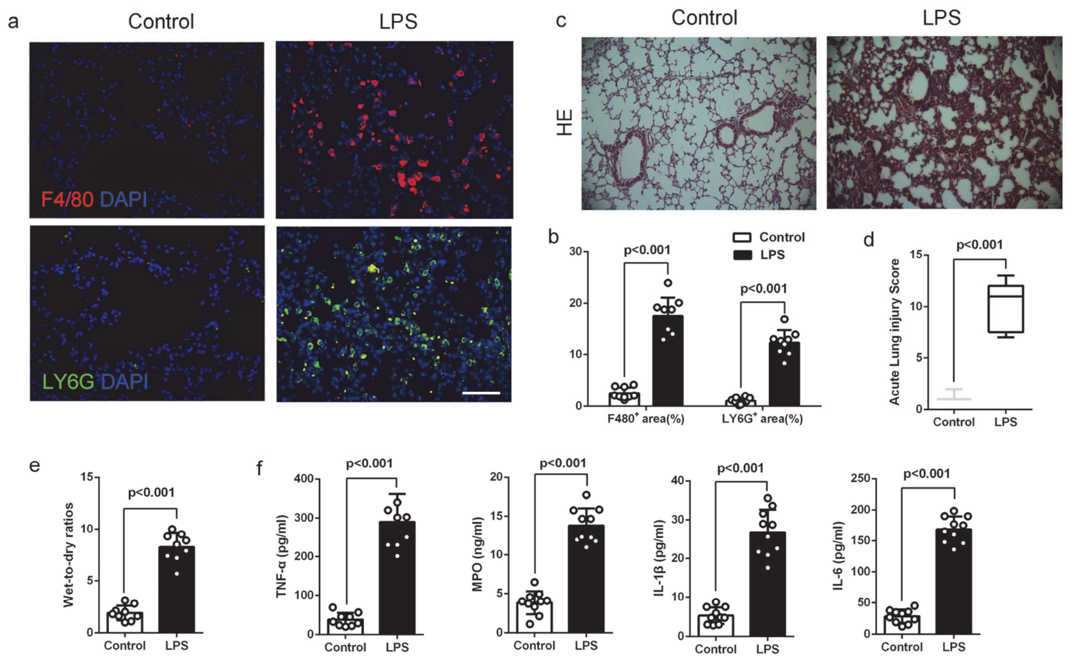

Supplemental Figure 7. LPS-induced sepsis mice had severe pulmonary edema and inflammatory response.

(a,b) Representative images and quantification of F4/80$^+$ cells (macrophages) and LY6G$^+$ cells (neutrophils) in lung sections (Control = 9, LPS = 9; representative data from three independent experiments). **(c,d)** Representative images of lung H&E-stained sections and the acute lung injury scores were determined by two independent investigators following the applicable criteria (Control = 9, LPS = 9; representative data from three independent experiments). **(e)** Wet-to-dry ratios for lungs (Control = 9, LPS = 9; representative data from three independent experiments). **(f)** The concentrations of inflammatory factors in lung tissue homogenate, such as TNF-α, MPO, IL-1β, and IL-6, were measured by ELISA (Control = 10, LPS = 10; representative data from three independent experiments). All n values refer to the number of mice used and the error bars depict mean±SD. p values were calculated by two-tailed unpaired Student's t-test. Only the lung injury scores were shown as Median (Quartile) [M (P25, P75)] and analyzed by using the Kruskal-Wallis test.

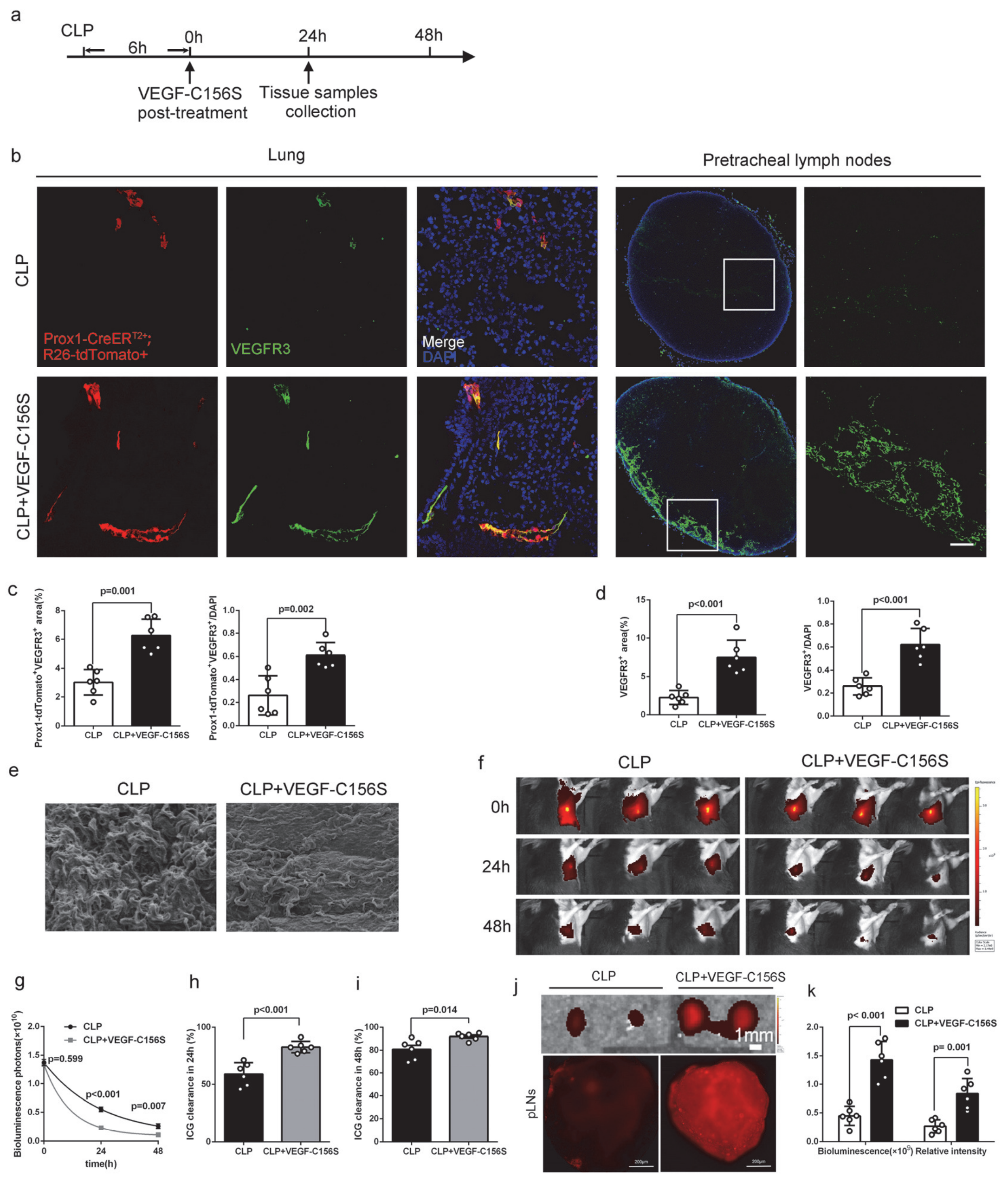

Supplemental Figure 8: Post-treatment of VEGF-C156 ameliorated pulmonary lymphatic drainage function through rejuvenating lymphatics in CLP-induced sepsis.

(a) Procedure and timeline: Recombinant VEGF-C156S protein was administrated to LPS-induced sepsis model 6 hours post, then, the lung tissue and the pretracheal lymph nodes (pLNs) were obtained at 24th hour. **(b)** Lymphatic vessels were labeled with immunofluorescence stain of VEGFR3 (green) and Prox1-tdTomato signals (red). Scale bars, 50 μm. **(c,d)** Quantification of the percent area coverage and the relative

fluorescence intensity of lymphatic vessels (CLP =6, CLP + VEGF-C156S =6; representative data from three independent experiments). **(e)** Scanning electron microscopy of the lymphatic endothelium in pulmonary lymphatic vasculature. Scale bars, 1μm. **(f,g)** ICG was intratracheally poured into unilateral lung and quantified and presented as relative radiance at 0th hour, 24th hour and 48th hour using IVIS. **(h,i)** The ICG clearance rate at 24th hour and 48th hour (CLP =6, CLP + VEGF-C156S =6; representative data from three independent experiments). **(j,k)** At 24th hour, ICG was intratracheally poured into unilateral lung in LPS-induced sepsis model. Fluorescence intensities of ICG were determined in pLNs at 30 minutes after the pour by IVIS and confocal microscopy (CLP n=6, CLP + VEGF-C156S n=6; representative data from three independent experiments). All n values refer to the number of mice used and the error bars depict mean ± SD. p values were calculated by two-tailed paired or unpaired Student's t-test.

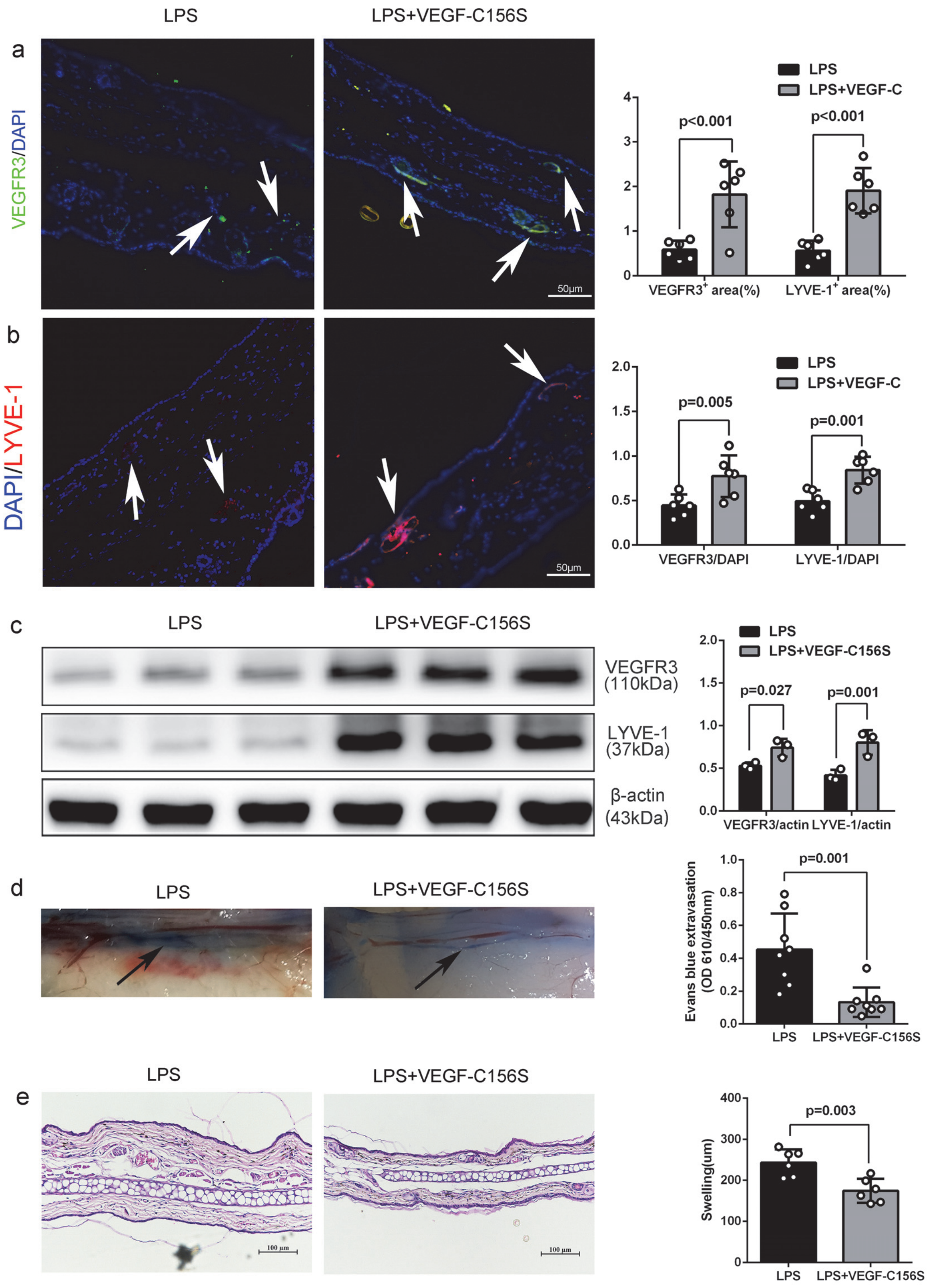

Supplemental Figure 9: Post-treatment of VEGF-C156S reduced the sepsis-induced edema through repairing the lymphatic vessel.

At 24th hour after VEGF-C156S administration, the skin tissues were obtained. **(a,b)** Representative images of ear dermal lymphatic vessels and quantification of the percent area coverage and the relative intensity of VEGFR3 or LYVE-1 staining (LPS =

6, LPS + VEGF-C156S = 6; representative data from three independent experiments). **(c)** VEGFR3 and LYVE-1 protein levels in skin tissue were detected by western blot (LPS = 3, LPS + VEGF-C156S = 3; representative data from three independent experiments). **(d)** Evans Blue was injected into the footpad and left circulating for 30 minutes. Quantification of formamide-extracted Evans Blue (LPS = 8, LPS + VEGF-C156S = 8; representative data from three independent experiments). **(e)** Representative images of ear skin H&E-stained sections. The severities of swelling were scored by the thickness of ears (LPS = 6, LPS + VEGF-C156S = 6; representative data from three independent experiments). All n values refer to the number of mice used and the errors bars depict mean ± SD. p values were calculated by two-tailed unpaired Student's t-test.

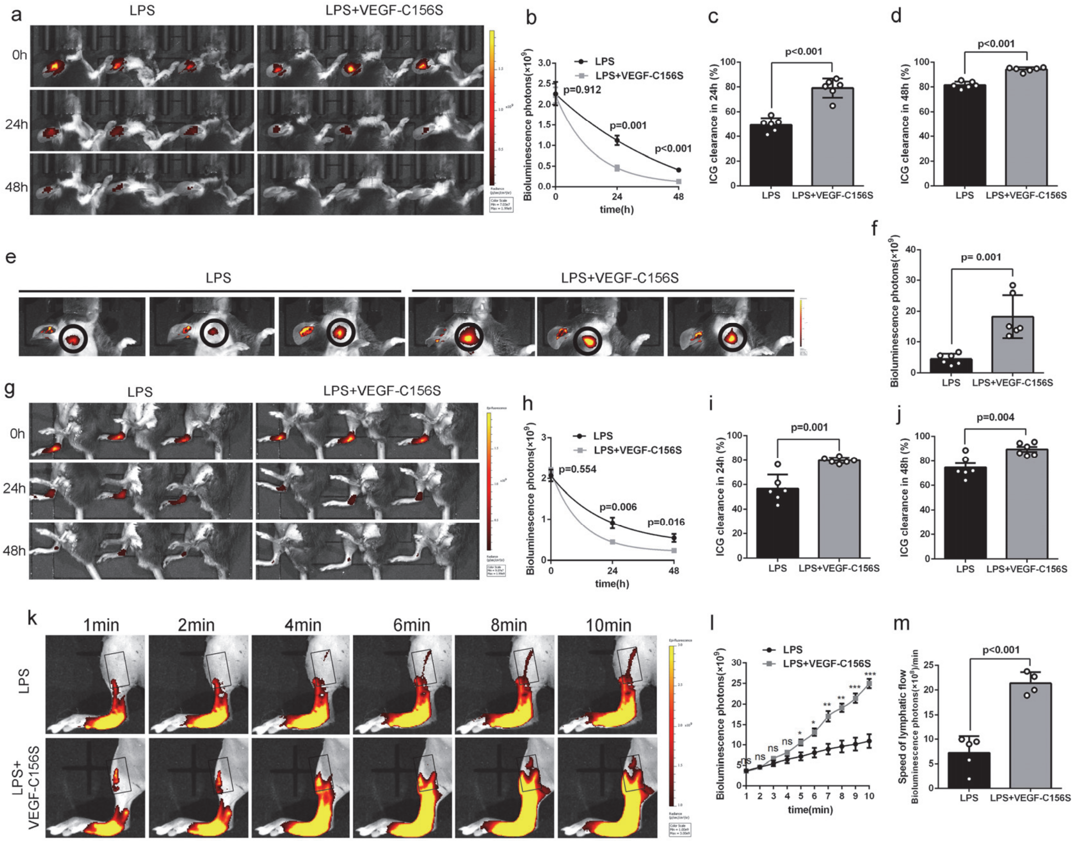

Supplemental Figure 10: Post-treatment of VEGF-C156S ameliorated dermal

lymphatic drainage function in LPS-induced sepsis.

(a-d) ICG was intradermally injected into the skin of the ear. Fluorescence intensities were determined at 0th hour, 24th hour and 48th hour using IVIS associated with the clearance rate to reflect the effect of tissue fluid clearance at 24th hour and 48th hour by lymphatic flow (LPS = 6, LPS + VEGF-C156S = 6; representative data from three independent experiments). **(e-f)** At 24th hour after VEGF-C156S administration, ICG was intradermally injected into the skin of ear. Fluorescence intensities were determined in the deep cervical lymph nodes (dcLNs) at 30 minutes after the injection and quantified quantified by IVIS (LPS = 6, LPS + VEGF-C156S = 6; representative data from three independent experiments). **(g-j)** ICG was intradermally injected into the footpad and quantified by IVIS. The ICG clearance rate at 24th hour and 48th hour (LPS = 6, LPS + VEGF-C156S = 6; representative data from three independent experiments). **(k-m)** ICG was detected in the lymphatic vessel (frame) within 10 minutes after footpad injection. The speed of lymphatic flow was determined by assessing pulses in the ICG signal intensity in the region of interest, which was graphed over time during the imaging session (LPS = 5, LPS + VEGF-C156S = 4; representative data from three independent experiments). All n values refer to the number of mice used and the errors bars depict mean ± SD. p values were calculated by two-tailed paired or unpaired Student’s t-test.

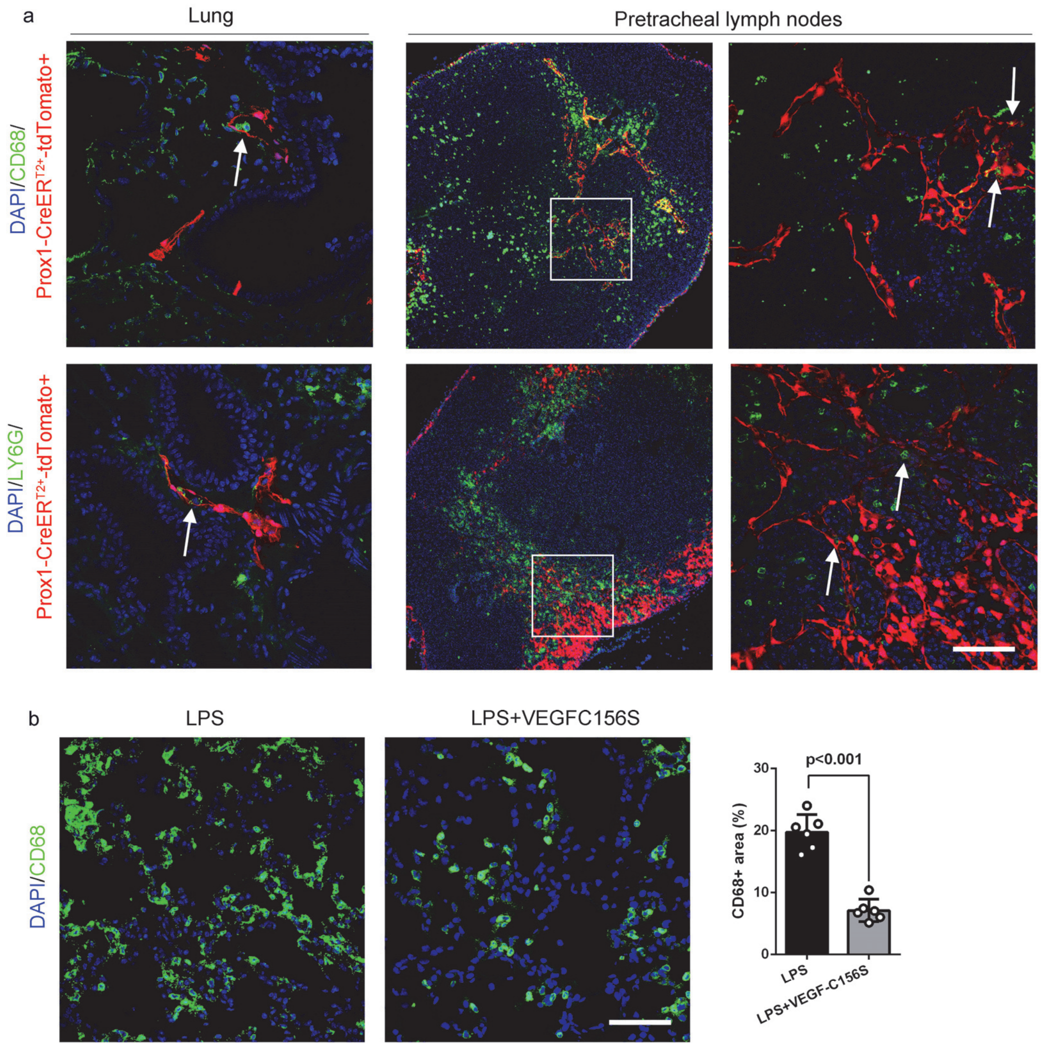

Supplemental Figure 11: VEGF-C156S post-treatment reduced the accumulation of pulmonary macrophages through lymphatic drainage during sepsis.

(a) Recombinant VEGF-C156S protein was administrated to LPS-induced sepsis model. Thereafter, the lung tissue and the pretracheal lymph nodes (pLNs) were obtained at 24th hour. Representative immunofluorescence images of $CD68^{+}$ cells (macrophages) and $LY6G^{+}$ cells (neutrophil) in lymphatic vessels (Prox1-$tdTomato^{+}$). Scale bars, 50 μm. **(b)** Representative immunofluorescence images and quantification of $CD68^{+}$ cells (green, macrophages) in lung sections (LPS = 6, LPS + VEGF-C156S = 6; representative data from three independent experiments). Scale bars, 50 μm. All n values refer to the number of mice used and the errors bars depict mean ± SD. p values were calculated by two-tailed paired or unpaired Student's t-test.

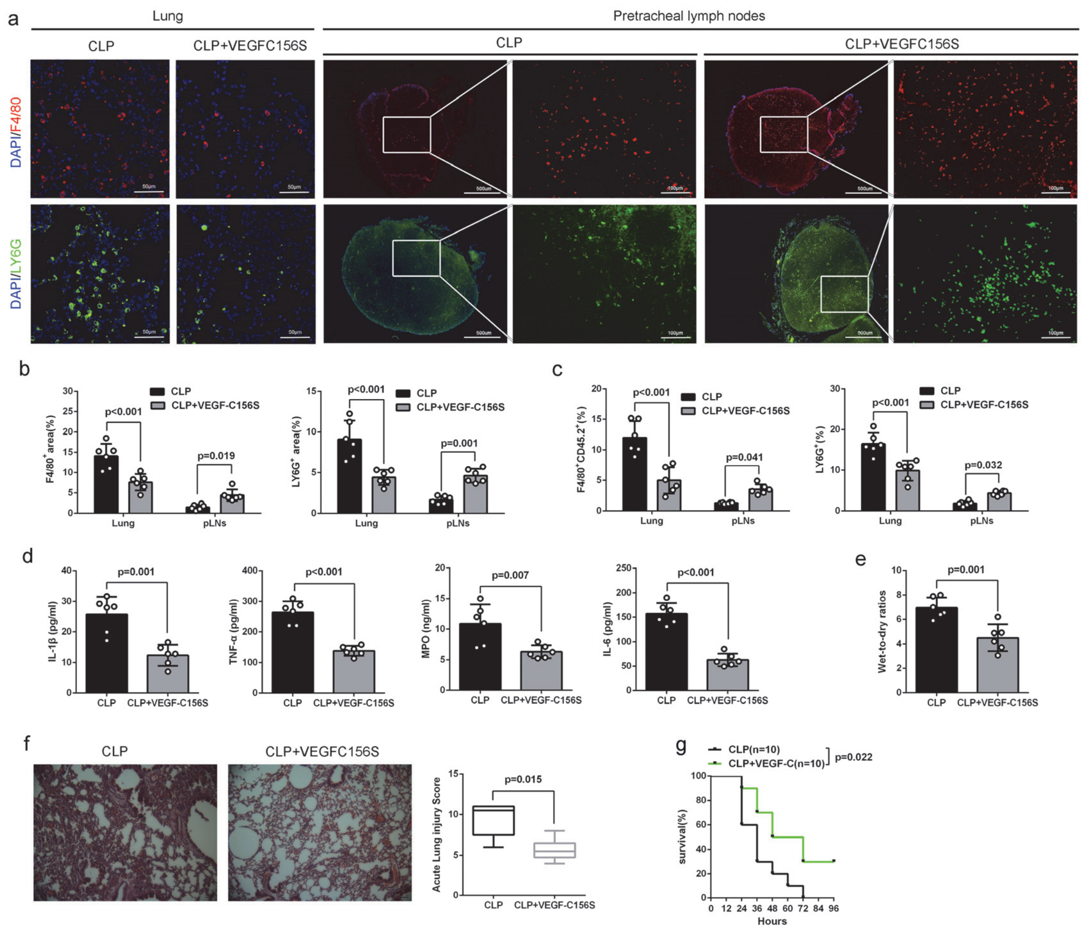

Supplemental Figure 12: VEGF-C156S post-treatment promoted pulmonary inflammatory cells to drain to pLNs in CLP-induced sepsis.

Recombinant VEGF-C156S protein was administrated to CLP-induced sepsis, then, the lung tissue and the pretracheal lymph nodes (pLNs) were obtained 24 hours later. **(a)** Representative immunofluorescence images of F4/80$^+$ cells (red, macrophages) or LY6G$^+$ cells (green, neutrophil) in lung sections and pLNs sections. **(b)** Quantification of F4/80$^+$ cells or LY6G$^+$ cells in lung sections and pLNs sections (CLP =6, CLP+VEGF-C156S =6; representative data from three independent experiments). **(c)** The percentage of F4/80$^+$/CD45.2$^+$ cells or LY6G$^+$ cells from the lung and pLNs by flow cytometry (CLP =6, CLP+VEGF-C156S =6; representative data from three independent experiments). **(d)** Representative images of lung H&E-stained sections and the acute lung injury scores (CLP =6, CLP + VEGF-C156S =6; representative data from three independent experiments). **(e)** Wet-to-dry ratios for lungs (CLP =6, CLP + VEGF-C156S =6; representative data from three independent experiments)**. (f)** The

concentrations of inflammatory factors in lung tissue homogenate, such as IL-1β, TNF-α, MPO, and IL-6, were measured by ELISA (CLP =6, CLP + VEGF-C156S =6; representative data from three independent experiments). **(g)** The roles of VEGF-C156S on survival rate in high-grade CLP-induced sepsis model. Mice received a single intraperitoneal injection with the operation of high-grade cecal ligation and puncture, followed by a single tail vein injection of VEGF-C156S (0.1 μg/g of body weight, 6 hours apart; CLP n=10, CLP+ VEGF-C156S n=10). All n values refer to the number of mice used and the errors bars depict mean ± SD. p values were calculated by two-tailed unpaired Student's t-test. The lung injury scores were shown as Median (Quartile) [M (P25, P75)] and analyzed by using the Kruskal-Wallis test. Survival in each subgroup was estimated by Kaplan-Meier survival curves and compared by the pairwise log-rank test

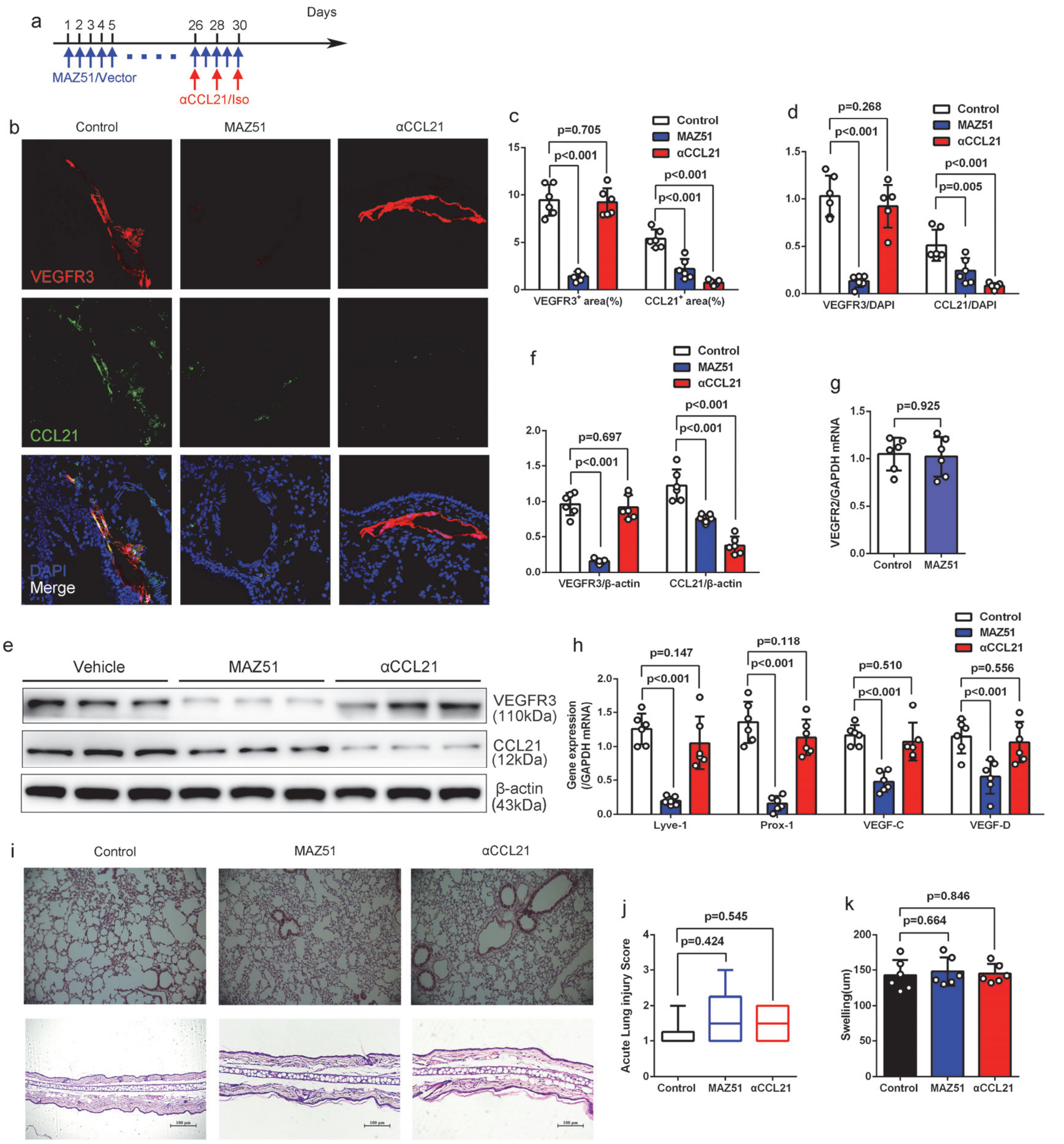

Supplemental Figure 13: Effect of the VEGFR3 inhibitor (MAZ51) or anti-CCL21 antibodies (αCCL21) on pulmonary lymphangiogenesis.

(a) Monitoring and treatment scheme. MAZ51 was intraperitoneally injected at 10 mg/kg of body weight for 30 days (5 days per week) for inhibiting VEGFR3. CCL21 was blocked on days 26, 28, and 30 by anti-CCL21 antibodies (αCCL21). **(b-d)** VEGFR3 (red) and CCL21 (green) were co-localized by immunofluorescence stain in lung sections. Quantification of the percent area coverage and the relative intensity of VEGFR3 and CCL21 staining. **(e-f)** VEGFR3 and CCL21 protein levels in lung tissue were detected by western blot. **(g)** The vascular markers VEGFR2 mRNA expression in lung tissue was detected by RT-PCR. **(h)** The lymphatic markers LYVE-1, Prox-1,

VEGF-C, and VEGF-D mRNA expression in lung tissue were detected by RT-PCR. **(i)** Representative images of the lung H&E-stained sections and the ear skin H&E-stained sections. **(j)** The acute lung injury scores. The lung injury scores were shown as Median (Quartile) [M (P25, P75)] and analyzed by using the Kruskal-Wallis test. **(k)** The severities of swelling were scored by the thickness of ears. Control n=6, MAZ51 n=6; αCCL21 n=6; representative data from three independent experiments. All n values refer to the number of mice used and the error bars depict mean ± SD. p values were calculated by a one-way ANOVA with Tukey's multiple comparison test.

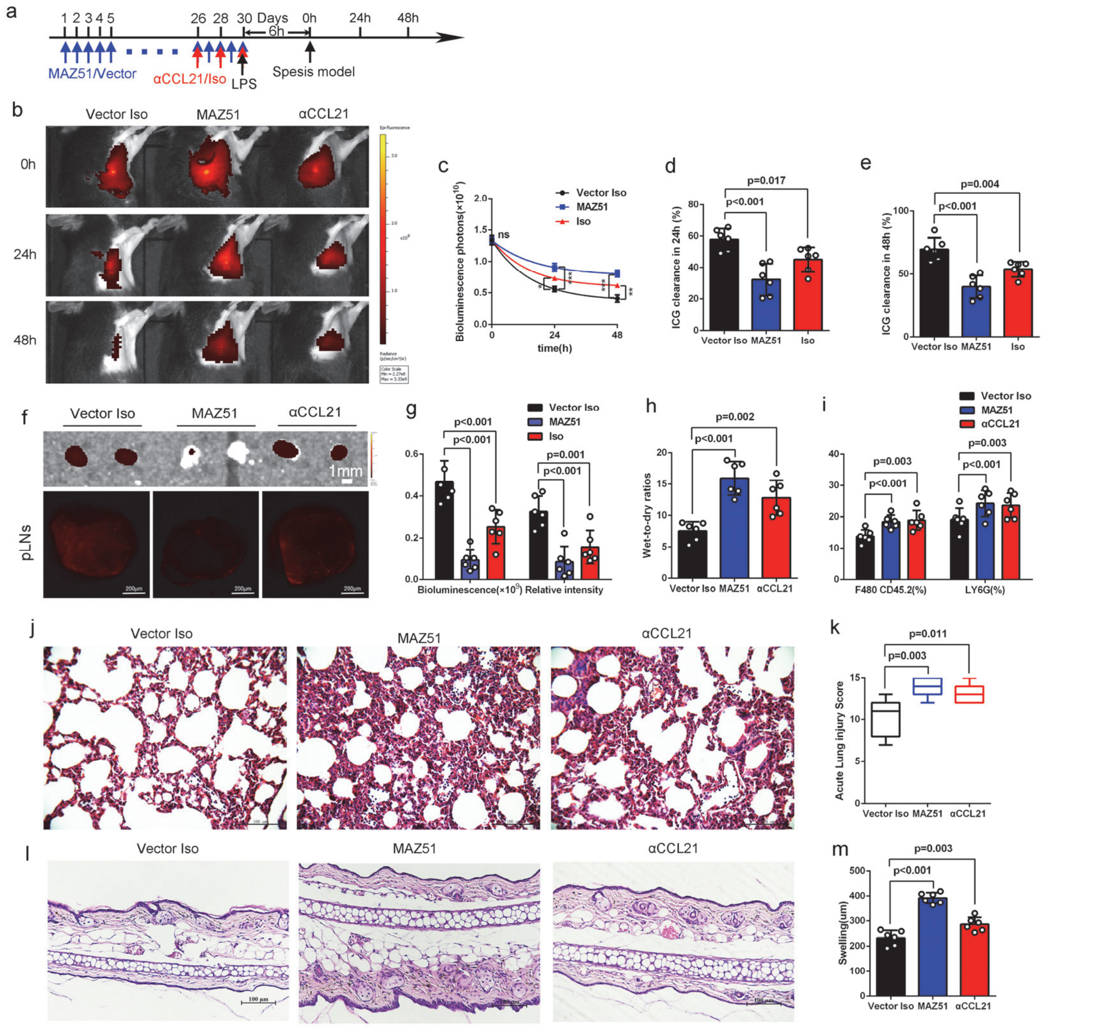

Supplemental Figure 14: Blocking VEGFR3 or CCL21 deteriorated

sepsis-induced lymphatic dysfunction and inflammation.

(a) Monitoring and treatment scheme. The sepsis model was induced by LPS intraperitoneally injection following the administration of MAZ51/Vector, anti-CCL21 (αCCL21) or IgG (Iso) antibodies. **(b-e)** The lymphatic drainage function was determined by IVIS. The remaining ICG was quantified and presented as relative radiance at 0th hour, 24th hour and 48th hour. The ICG clearance rate at 24th hour and 48th hour. **(f-g)** Fluorescence intensities of ICG were determined in mLNs at 30 minutes after the pour of ICG by IVIS and confocal microscopy at 24th hour. **(h)** Wet-to-dry ratios for lungs. **(i)** Quantification of $F4/80^{+}/CD45.2^{+}$ cells and LY6G+ cells in lung tissue by flow cytometry at 24th hour. **(j-k)** Representative images of lung H&E-stained sections and the acute lung injury scores showed as Median (Quartile) [M (P25, P75)] and analyzed by using the Kruskal-Wallis test. **(l-m)** Representative images of ear skin H&E-stained sections and the severities of swelling were scored by the thickness of ears. Vector Iso n=6, MAZ51 n=6; αCCL21 n=6; representative data from three independent experiments. All n values refer to the number of mice used and the error bars depict mean±SD. p values were calculated by a one-way ANOVA with Tukey's multiple comparison test.

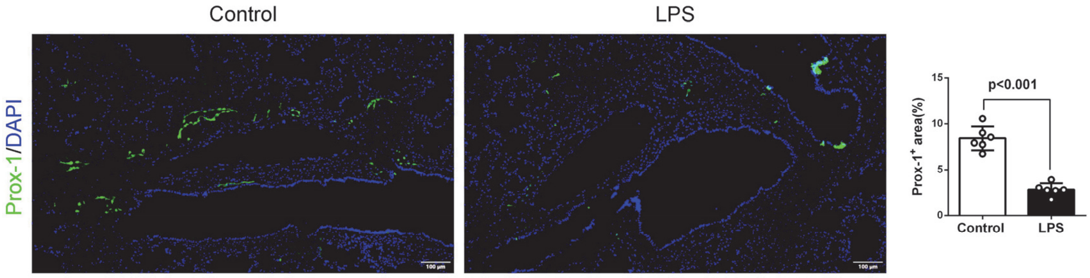

Supplemental Figure 15. The area of pulmonary lymphatic vessels was significantly reduced in sepsis mice.

At 24th hour, lungs were obtained from LPS-induced sepsis mice. Lung sections were subjected to immunofluorescence stain for Prox-1 (green) for lymphatic vessels. Representative images of lung lymphatic vessels and quantification of the percent area coverage of Prox-1 staining. (n=6 per group; representative data from three

independent experiments). All n values refer to the number of mice used and the error bars depict mean ± SD. p values were calculated by a one-way ANOVA with Tukey's multiple comparison test.

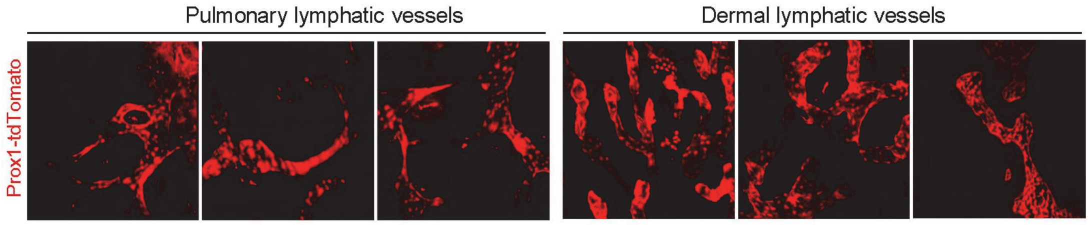

Supplemental Figure 16. Observing the lymphatic vessels by PROX1-tdTomato fluorescence.

The *Prox1-CreERT2;Rosa26-tdTomato* mice were treated with 50 mg/kg tamoxifen for four consecutive days by intraperitoneal injection. Four days after the last injection, the pulmonary lymphatic vessels and dermal lymphatic vessels were observed by wholemount microscopy.

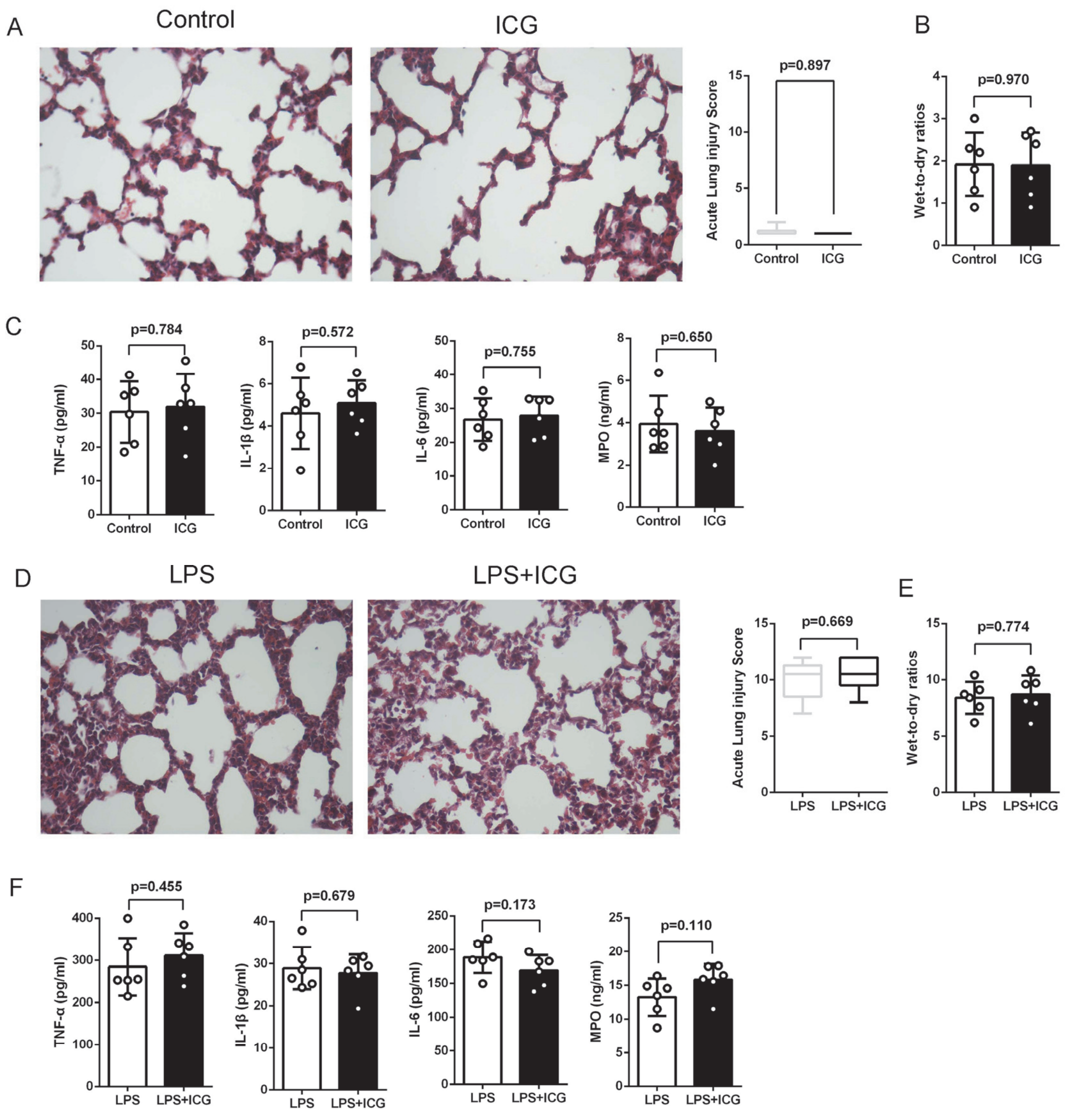

Supplemental Figure 17. Low-dose ICG does not cause or deteriorate lung injury during normal or septic state.

ICG (1 mg/ml, 10 µL) was intratracheally poured into the lung in control mice or sepsis mice (10 mg/kg LPS intraperitoneal injection), then, the lung tissue was obtained at 24th hour. (**A,D**) Representative images of lung H&E-stained sections and the acute lung injury scores. (**B,E**) Wet-to-dry ratios for lungs. (**C,F**) The concentrations of inflammatory factors in lung tissue homogenate, such as TNF-α, IL-1β, IL-6, MPO, and were measured by ELISA. All n values refer to the number of mice used and the error bars depict mean±SD. p values were calculated by two-tailed unpaired Student's t-test. The lung injury scores were shown as Median (Quartile) [M (P25, P75)] and analyzed by using the Kruskal-Wallis test. The survival rate in each subgroup was estimated by Kaplan-Meier survival curves and compared by the pairwise log-rank test.

.

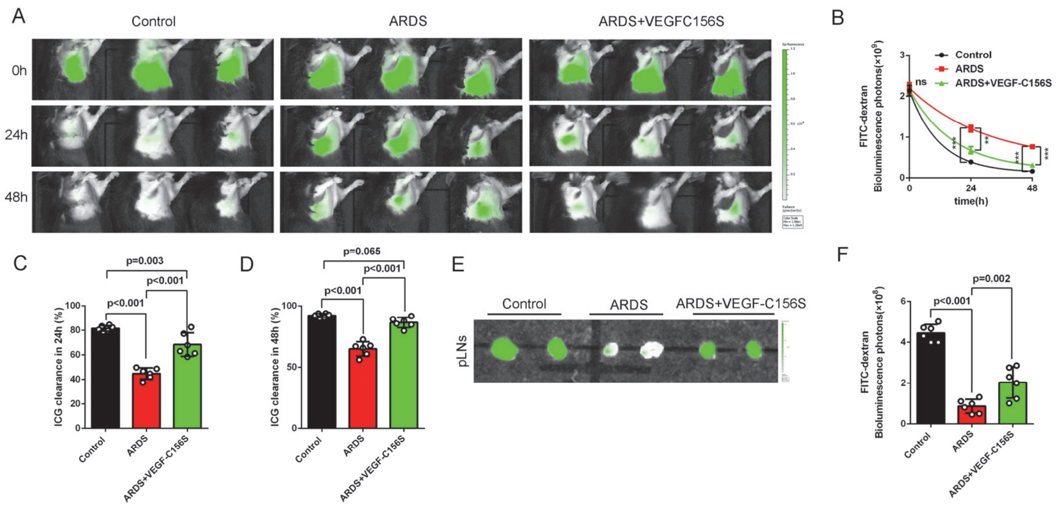

Supplemental Figure 18. VEGF-C156S ameliorated pulmonary lymphatic drainage function in sepsis-induced ARDS by using FITC-dextran IVIS.

(**A,B**) FITC-dextran (5 mg/ml, 10 μL) was intratracheally poured into unilateral lung and quantified and presented as relative radiance at 0th hour, 24th hour and 48th hour using IVIS. **(C,D)** The FITC-dextran clearance rate at 24th hour and 48th hour (n=6 per group; representative data from three independent experiments). **(E,F)** At 24th hour, FITC-dextran was intratracheally poured into unilateral lung. Fluorescence intensities of FITC-dextran were determined in pLNs at 30 minutes after the pour by IVIS and confocal microscopy (n=6 per group; representative data from three independent experiments). All n values refer to the number of mice used and the error bars depict mean ± SD. p values were calculated by a one-way ANOVA with Tukey's multiple comparison test.

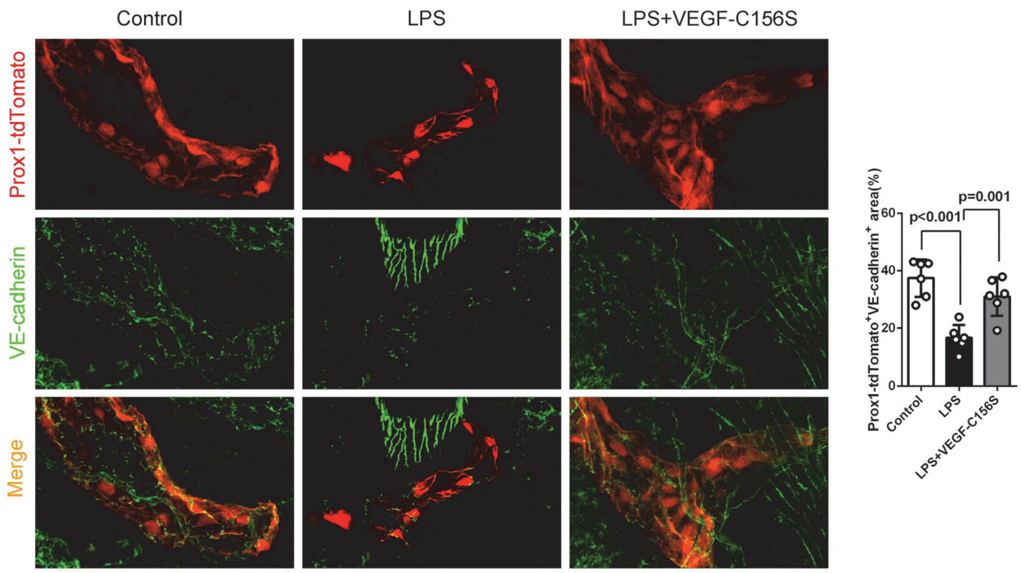

Supplemental Figure 19. VEGF-C156S ameliorates pulmonary lymphatic integrity in sepsis.

Lung sections were subjected to immunofluorescence stain for VE-cadherin (green) in lymphatic vessels (Prox1-tdTomato) by wholemount microscopy. Representative images of pulmonary lymphatic endothelial junction and quantification of the percent area coverage of Prox1-tdTomato^{+}VE-cadherin^{+} staining (n=6 per group; representative data from three independent experiments). All n values refer to the number of mice used and the error bars depict mean ± SD. p values were calculated by a one-way ANOVA with Tukey's multiple comparison test.

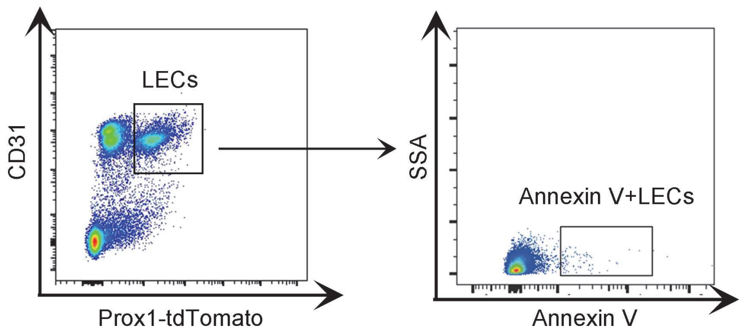

Supplemental Figure 20. Identification of dead LECs by flow cytometry.

Lymphatic endothelial cells (LECs) were isolated from lung suspension by CD31+Prox1tdtomato+ staining. Then, dead LECs were identify by Annexin V staining from LECs.

Supplementary Videos

Supplementary Video 1. The consecutive lymphatic flow in control mice. Shown is a lymphangiography performed immediately after TITC-dextran subcutaneous injection in which consecutive TITC-dextran flow could be seen in the superficial lymphatic vessels in Control mice.

Supplementary Video 2. The obstructed lymphatic flow in sepsis mice. Shown is a lymphangiography in which obstructed TITC-dextran flow could be seen in the superficial lymphatic vessels in sepsis mice injected with 10mg/kg LPS for 24 hr.

Supplementary Video 3,4. ICG drains primarily via the pulmonary lymphatic vessels in normal or sepsis mice.

Indocyanine Green (ICG, 1mg/mL, 5μL, green) was intratracheally poured into the lung of the *Prox1-CreERT2;Rosa26-tdTomato* mice. 30 min later, exposing the chest under the respiratory support with animal ventilator and detecting the pulmonary lymphatic drainage of normal or sepsis mice by multiphoton imaging system. Lymphatic vessels were labeled by Prox1-tdTomato (red) fluorescence.

Supplementary table

Supplementary Table 1. The sequences of the primers used.

Name	F/R	Sequences (5' to 3')
β-actin	F	GGCTGTATTCCCCTCCATCG
	R	CCAGTTGGTAACAATGCCATGT
VEGF-A	F	ACGACAGAAGGAGAGCAGAAG
	R	ATGTCCACCAGGGTCTCAATC
VEGF-C	F	CAGCCCACCCTCAATACCAG
	R	GCTGCTCCAAACTCCTTCC
VEGF-D	F	TGGCAAGACTTTTGAGCTTCAA
	R	AAATCGCGCACTCTGAGGA
Prox1	F	CAGCGGACTCTCTAGCACAG
	R	GCCTGCCAAAAGGGGAAAGA
Lyve1	F	CTGACAAGCAGTTTCAGGCTTGGT
	R	TTCAGCCCACACTCCGCTATACAT
VEGFR2	F	GTGACCAACATGGAGTCGTG
	R	CCAGAGATTCCATGCCACTT
VEGFR3	F	GCGACAGGGTTCTCATAA
	R	CGTTGCCTCATTGTGATTAG
Adgre1	F	CTGTAACCGGATGGCAAACT
	R	CTGTACCCACATGGCTGATG
CCl19	F	GGGGTGCTAATGATGCGGAA
	R	CCTTAGTGTGGTGAACACAACA
CCl21	F	CCCTGCTTCAACCATTACATCTGC
	R	CCTGCTGTCTCCTTCCTCATTCC
CCR7	F	TGTACGAGTCGGTGTGCTTC
	R	GGTAGGTATCCGTCATGGTCTTG
LY6G	F	GATGGATTTTGCGTTGCTCT
	R	TTGTCCAGAGTAGTGGGGCAGA
CD11c	F	CTGGATAGCCTTTCTTCTGCTG

	R	GCACACTGTGTCCGAACTC
CD3e	F	GATGCGGTGGAACACTTTCT
	R	ACTGTCCTCGACTTCCGAGA
Tbx21	F	AGCAAGGACGGCGAATGTT
	R	GGGTGGACATATAAGCGGTTC
Angptl7	F	TATCCACCGGCTCACCAG
	R	TCTGCGTAGCGTGCATTG
Angptl4	F	GGGACCTTAACTGTGCCAAG
	R	GAATGGCTACAGGTACCAAACC
